# Endophilin A and B Join Forces With Clathrin to Mediate Synaptic Vesicle Recycling in *Caenorhabditis elegans*

**DOI:** 10.3389/fnmol.2018.00196

**Published:** 2018-06-14

**Authors:** Szi-chieh Yu, Barbara Jánosi, Jana F. Liewald, Sebastian Wabnig, Alexander Gottschalk

**Affiliations:** ^1^Buchmann Institute of Molecular Life Sciences (BMLS), Goethe University, Frankfurt, Germany; ^2^Institute of Biophysical Chemistry, Department of Biochemistry, Chemistry and Pharmacy, Goethe University, Frankfurt, Germany; ^3^Cluster of Excellence Frankfurt, Macromolecular Complexes (CEF-MC), Goethe University, Frankfurt, Germany

**Keywords:** endophilin, clathrin heavy chains, synaptic vesicles, recycling pathway, optogenetic stimulation, electron microscopy, electrophysiology, genetic analysis

## Abstract

Synaptic vesicle (SV) recycling enables ongoing transmitter release, even during prolonged activity. SV membrane and proteins are retrieved by ultrafast endocytosis and new SVs are formed from synaptic endosomes (large vesicles—LVs). Many proteins contribute to SV recycling, e.g., endophilin, synaptojanin, dynamin and clathrin, while the site of action of these proteins (at the plasma membrane (PM) vs. at the endosomal membrane) is only partially understood. Here, we investigated the roles of endophilin A (UNC-57), endophilin-related protein (ERP-1, homologous to human endophilin B1) and of clathrin, in SV recycling at the cholinergic neuromuscular junction (NMJ) of *C. elegans*. *erp-1* mutants exhibited reduced transmission and a progressive reduction in optogenetically evoked muscle contraction, indicative of impaired SV recycling. This was confirmed by electrophysiology, where particularly endophilin A (UNC-57), but also endophilin B (ERP-1) mutants exhibited reduced transmission. By optogenetic and electrophysiological analysis, phenotypes in the *unc-57; erp-1* double mutant are largely dominated by the *unc-57* mutation, arguing for partially redundant functions of endophilins A and B, but also hinting at a back-up mechanism for neuronal endocytosis. By electron microscopy (EM), we observed that *unc-57* and *erp-1; unc-57* double mutants showed increased numbers of synaptic endosomes of large size, assigning a role for both proteins at the endosome, because endosomal disintegration into new SVs, but not formation of endosomes were hampered. Accordingly, only low amounts of SVs were present. Also *erp-1* mutants show reduced SV numbers (but no increase in LVs), thus ERP-1 contributes to SV formation. We analyzed temperature-sensitive mutants of clathrin heavy chain (*chc-1*), as well as *erp-1; chc-1* and *unc-57; chc-1* double mutants. SV recycling phenotypes were obvious from optogenetic stimulation experiments. By EM, *chc-1* mutants showed formation of numerous and large endosomes, arguing that clathrin, as shown for mammalian synapses, acts at the endosome in formation of new SVs. Without endophilins, clathrin formed endosomes at the PM, while endophilins A and B compensated for the loss of clathrin at the PM, under conditions of high SV turnover.

## Introduction

**Figure 1:**
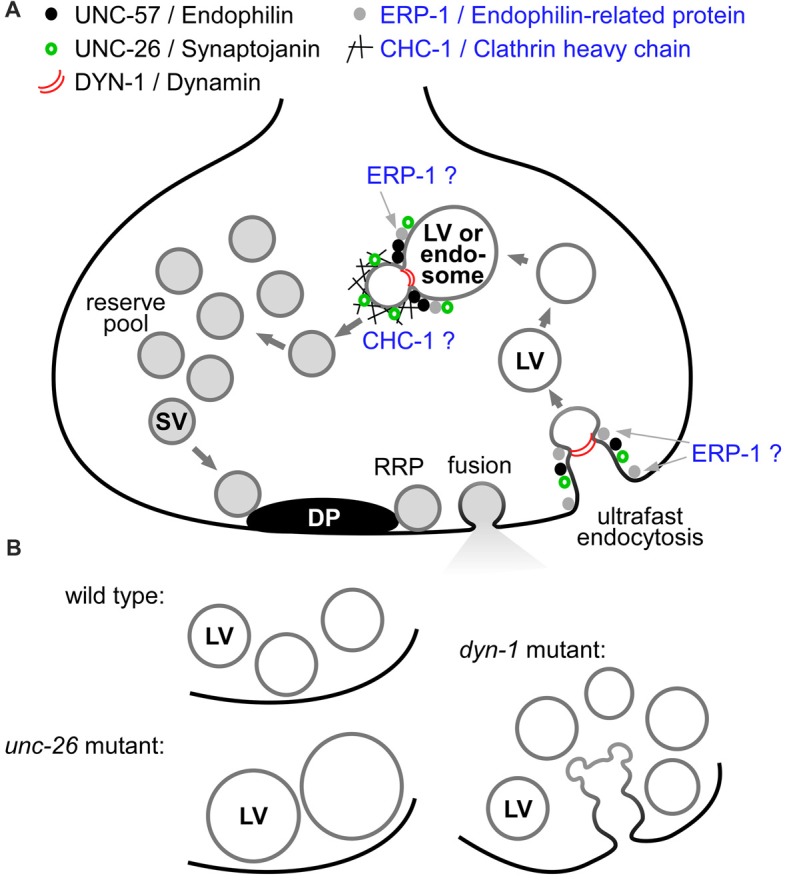
Model of the synaptic vesicle (SV) cycle and select proteins involved in distinct steps of SV recycling. **(A)** SVs are formed by budding from synaptic endosomes, entering a reserve pool (RP) after filling with neurotransmitter (gray lumen). At the dense projection (DP), SVs approach the plasma membrane (PM; black line) and become docked and primed, forming the readily releasable pool (RRP). After fusion, ultrafast endocytosis retrieves the SV membrane and proteins, forming clear-core large vesicles (LVs), following membrane severing by dynamin (red arcs). The LVs fuse with or transform into synaptic endosomes. Several proteins studied either previously (black dots: UNC-57/endophilin A, green open circles: UNC-26/synaptojanin, red arcs: DYN-1/dynamin; Kittelmann et al., 2013), or as part of this work (black mesh: CHC-1/clathrin heavy chain, gray dots: ERP-1/endophilin B), affect different steps of the recycling pathway, acting either at the PM or at the synaptic endosome. Roles/sites of action of clathrin and endophilin-related protein (ERP-1; blue text) are not firmly established as yet in *C. elegans*. **(B)** Summary of mutant phenotypes as observed at the ultrastructural level, following photostimulation of cholinergic neurons (Kittelmann et al., 2013): in wild type, LVs form transiently. In the *unc-26* mutant, less LVs form, but they do form and are persistent, as they cannot be efficiently broken down. In the *dyn-1* mutant, LVs are formed but cannot be pinched off the PM, and smaller vesicles emerge from these structures (possibly new SVs) that can also not be pinched off. LVs that were formed nonetheless are accumulating and cannot be processed to SVs efficiently.

Synaptic transmission is orchestrated by intricate protein machinery (Sudhof, 2013). Synaptic vesicles (SVs) are synthesized in the synaptic terminal, from components (membrane, proteins) delivered from the cell soma (Hannah et al., 1999). These components are thought to be assembled in the synaptic endosome and packaged into uniform vesicles by the clathrin-associated machinery, as shown for mammalian synapses following optogenetic stimulation (Watanabe et al., 2014); see Figure 1A for a model. SVs are filled with neurotransmitter, through the action of the vesicular ATPase and specific, proton-driven neurotransmitter transporters, like the vesicular acetylcholine (ACh) transporter. They then enter the so-called reserve pool (RP) of vesicles. From the RP, they can be mobilized in an activity-dependent manner, and translocate towards the plasma membrane (PM). Specific protein machinery at the center of the active zone, called presynaptic particle web, cytomatrix, or dense projection (DP), depending on the organism and synapse type, is believed to guide the SV towards the PM, and to prepare contacts between PM proteins of the fusion machinery and proteins of the SV (Sudhof, 2012). The vesicle undergoes docking and priming, after which the SV is in a state which only requires a sharp rise in the cytosolic Ca^2+^ concentration, in order to execute the fusion in sub-millisecond time scale. Depolarization and voltage-gated Ca^2+^ channels (VGCCs) provide this signal. Following fusion, the process of ultrafast endocytosis retrieves the SV membrane and its protein constituents. This form of endocytosis (also called bulk endocytosis) occurs within 50–100 ms after fusion, and at physiological temperature is independent of clathrin (Kittelmann et al., 2013; Watanabe et al., 2013a,b, 2014; Soykan et al., 2017). It leads to the formation of clear-core synaptic endosomes (Jahne et al., 2015), also called “large vesicles (LVs)” or “100 nm vesicles,” from which SVs are reformed within about 10 s; the latter process is clathrin-dependent (Kittelmann et al., 2013; Watanabe et al., 2014). However, the precise sequence of events and proteins involved in SV recycling is still under debate.

Numerous proteins affect the rate and effectivity of SV recycling (Dittman and Ryan, 2009; Haucke et al., 2011; Kononenko et al., 2014; Rizzoli, 2014; Kononenko and Haucke, 2015). Synaptojanin and endophilin are required for clathrin-mediated endocytosis (CME), and are also crucial for SV recycling (Harris et al., 2000; Verstreken et al., 2002; Schuske et al., 2003; Dickman et al., 2005; Liewald et al., 2008; Kittelmann et al., 2013; Wabnig et al., 2015). However, given that two stages of the SV recycling process require vesicle formation, one at the PM, and one at the endosome, determining their precise site of action is an important task. Synaptojanin is required for vesicle uncoating, following CME (Verstreken et al., 2003). For endophilin, several functions have been determined. It is required for binding to and bending membranes at sites of endocytosis, and for recruitment of dynamin and synaptojanin to the budding membrane. Thus, it is expected that also ultrafast endocytosis, which requires the severing of the endocytosed membrane by dynamin, requires endophilin function. This has been demonstrated to some extent: in addition to its role in CME, endophilin A2 is also required for clathrin-independent endocytosis (CIE) in non-neuronal cells, where it acts in parallel/in addition to dynamin for membrane shaping and scission (Boucrot et al., 2015; Renard et al., 2015). This mechanism is likely analogous to the process otherwise referred to as (activity-dependent) bulk endocytosis (ADBE) in neurons, which in turn may represent the “end-product” of sustained ultrafast endocytosis (Cousin, 2017). CIE requires endophilin A2, cargo receptors, dynamin and actin polymerization, as shown in non-neuronal cells (Boucrot et al., 2015), and similar requirements were found for ultrafast endocytosis at neuronal synapses (Kononenko et al., 2014; Watanabe et al., 2014). One study suggested that the main function of endophilin is to recruit synaptojanin, because CME still occurred in endophilin A triple knockout mice, but coated vesicles accumulated (Milosevic et al., 2011). Recent work in *C. elegans* showed that only the membrane bending function of UNC-57/endophilin A is required to enable synaptic transmission. Transmission was also (partially) maintained when UNC-57 was tethered to the PM, indicating that it must act at the PM, while full functionality may require its presence also at the endosome (Bai et al., 2010). This group also characterized the interaction of UNC-57/endophilin A and UNC-26/synaptojanin; since they found identical phenotypes in the two mutants, they argued that both act in precisely the same biological pathways (Dong et al., 2015). Further work in *C. elegans* suggested, however, that they may be part of slightly different pathways (Kittelmann et al., 2013). Endophilin A and synaptojanin were not absolutely required for the ultrafast/bulk endocytosis step at the PM, because in *unc-26* (encoding synaptojanin) and *unc-57* mutants, LVs/endosomes were still formed after optogenetic stimulation. At the same time, the breakdown of these LVs into new SVs was strongly attenuated in both *unc-26* and *unc-57* mutants, arguing that the two proteins act at the step of SV formation from the endosome. Here, however, the *unc-26* phenotype was more severe, resulting in extremely large, persistent LVs (Kittelmann et al., 2013; summarized in Figure 1B). In fact, these mutants accumulate LVs even without optogenetic stimulation, simply due to normal synaptic activity. Particularly human endophilin A1 is attributed a major function in SV endocytosis, however, there is also endophilin B1, whose function is less well understood. Most work on vertebrate endophilin B suggests functions in intracellular membranous compartments, like autophagosomes, mitochondria and early endosomes, where it appears to be involved in sorting of PM receptors for recycling, away from the late endosomal/lysosomal pathway (Farsad et al., 2001; Modregger et al., 2003; Wan et al., 2008; Kjaerulff et al., 2011). In *C. elegans*, endophilin B is encoded by endophilin-related protein (ERP-1; see Supplementary Figure S1 for an alignment of these proteins).

The GTPase dynamin is required for severing of the membranes, both at the PM-LV transition as well as at the endosome-SV transition (Sever et al., 2000; Hayashi et al., 2008; Carpentier et al., 2013; Gormal et al., 2015). In agreement with this, *dyn-1* (encoding dynamin in *C. elegans*) mutants accumulate endocytosis intermediates at the PM, from which budding of new SVs can be observed; at the same time, disintegration of endosomes is delayed (Kittelmann et al., 2013). Thus, dynamin acts at both stages of the recycling process (Figure 1). For clathrin, work in mammalian synapses demonstrated a role at the endosome-SV transition, and showed that it is not required for ultrafast endocytosis (Watanabe et al., 2014). The authors also established that clathrin becomes crucial for endocytosis under non-physiological conditions, i.e., temperatures below 37°C, when classical CME is utilized as a safeguard mechanism that is too slow, though, to maintain sufficient SV recycling at high neuronal firing rates. For *C. elegans*, even though a role of clathrin in SV biogenesis has been deduced, it has not been demonstrated at which stage in the SV cycle it is acting (Sato et al., 2009). Thus, this was one of the aims of the present work, i.e., to use electron microscopy (EM) and optogenetic stimulation to identify the likely site of clathrin action (Figure 1A). Likewise, we wanted to analyze a possible role of ERP-1 in SV recycling in more detail, because we had identified *erp-1* in a screen designed to uncover SV recycling genes (Wabnig et al., 2015).

Here, we used a combination of pharmacological, behavioral, optogenetic, electrophysiological and ultrastructural assays to probe the roles of UNC-57, ERP-1 and of clathrin heavy chain (CHC-1) in SV recycling in *C. elegans*. Mutants lacking ERP-1 have reduced cholinergic transmission, and exhibited a progressive decline in SV exocytosis following cholinergic neuron long-term photostimulation. The *unc-57*/endophilin A; *erp-1*/endophilin B double mutant showed much stronger defects, however, those were dominated by the lack of *unc-57*, and no exacerbation of the *unc-57* phenotype was obvious in the double mutant. By EM analyses, *erp-1* mutants (after stimulation) exhibited enhanced formation of LVs, the end-product of (sustained) ultrafast endocytosis. Thus, ERP-1 appears to act in a partially redundant manner with UNC-57 at the stage of breakdown of synaptic endosomes to form new SVs, but is dispensable for bulk/ultrafast endocytosis. For CHC-1, we show that temperature-sensitive mutants also exhibit a SV recycling phenotype in behavioral assays, and that they accumulate unusually large LVs. Thus, as in mammals, *C. elegans* clathrin is not essential for bulk/ultrafast endocytosis, but acts mainly in de-novo generation of SVs at the synaptic endosome. To assess whether the remaining SV recycling in (*erp-1* and) *unc-57* mutants could be due to the function of CHC-1, we analyzed *erp-1; chc-1* and *unc-57; chc-1* double mutants. Our data suggest mutually redundant functions of the three proteins in SV recycling, with the strongest contribution by endophilin A/UNC-57. Loss of function can be partially compensated by each of these proteins, when the respective other two proteins are missing, thus enabling sufficient activity to maintain synaptic transmission and survival.

## Materials and Methods

### Nematode Strains and Genetics

The wild-type *C. elegans* Bristol strain N2 was used for some experiments and cultivated as previously described (Brenner, 1974). Also used or generated in this study were the following mutant strains: **CB406**: *unc-57(e406)I*, **DH1230**: *chc-1(b1025ts)III*, **RB700**: *erp-1(ok462)X*, **ZX2088**: *erp-1(ok462)X; unc-57(e406)I*. The following transgenic strains were generated and/or used: **ZX460**: wild type; *zxIs6[punc-17::ChR2(H134R)::YFP; lin-15+]V* (Liewald et al., 2008), **ZX634**: *unc-57(e406)I; zxIs6[punc-17::ChR2(H134R)::YFP; lin-15+]V*, **ZX1602**: *erp-1(ok462)X; zxIs6[punc-17::ChR2(H134R)::YFP; lin-15+]V*, **ZX1778**: *chc-1(b1025ts)III; zxIs6[punc-17::ChR2(H134R)::YFP; lin-15+]V*, **ZX2227**: *unc-57(e406)I; erp-1(ok462)X; zxIs6[punc-17::ChR2(H134R)::YFP; lin-15+]V*, **ZX2342**: *chc-1(b1025)III; unc-57(e406)I; zxIs6[punc-17::ChR2(H134R)::YFP; lin-15+]V*, **ZX2343**: *chc-1(b1025)III; erp-1(ok462)X; zxIs6[punc-17::ChR2(H134R)::YFP; lin-15+]V*, **BC12478**: *dpy-5(e907)I; sIs10597[rCes F35A5.8a::GFP + pCeh361]* (McKay et al., 2003).

### Fluorescence Microscopy

Expression of p*erp-1*::GFP (strain BC12478) and p*unc-17*::ChR2(H134R)::YFP (strain ZX460) were analyzed on a Zeiss Axiovert 200 microscope (Zeiss, Germany), with a 40×/0.25 Zeiss oil objective and GFP filter set (Ex 470/40 nm, Em 525/50 nm). Micrographs were taken with an ORCA-Flash4.0 Digital sCMOS Camera (Hamamatsu Photonics, Japan). Animals were transferred onto 10% agarose pads in M9 buffer (K_2_PO_4_ 20 mM; Na_2_HPO_4_ 40 mM; NaCl 80 mM; MgSO_4_ 1 mM) and immobilized with Polybead polystyrene 0.1 mm microspheres (Polysciences Inc., Warrington, PA, USA).

### Behavioral Experiments

Transgenic worms were cultivated in the dark at 20°C on nematode growth medium (NGM) dishes with OP50 bacteria (Brenner, 1974) without or with all-trans retinal (ATR; Liewald et al., 2008). Dishes containing ATR were prepared by spreading 320 μl of OP50 culture mixed with 0.64 μl of 100 mM ATR stock (dissolved in ethanol) onto 5.5-cm dishes containing 8.2 ml of NGM. About 18 h before experiments, L4 larvae, grown on ATR plates, were placed on fresh ATR plates. Worms were illuminated with blue light (contraction-assay: 1.4 mW/mm^2^, swimming-assay: 0.53 mW/mm^2^) from a 50 W mercury lamp, filtered through a GFP excitation filter (450–490 nm), on 5.5 cm diameter dishes, under a 10× objective in a Zeiss Axiovert40 microscope (Zeiss, Germany). Duration of illumination was defined by a computer-controlled shutter (Sutter Instruments). Worms were filmed with a Powershot G9 digital camera (Canon, Japan) at 640 × 480 resolution with 30 fps. Body length was determined as previously described (Erbguth et al., 2012). The length values were normalized to the averaged values measured before illumination (0–4 s), normalization was carried out for each worm. All the values below 80% were excluded and the length-profiles were averaged for each strain. The experiments were repeated on 2–3 different days (worms were picked from different populations on different days); the final graphs show the average of all individual animals. For the heat-shock (hs) experiments worms were cultivated at 15°C. The length of the hs was 14 min at 30°C, worms were picked to pre-warmed (at least 3 h in 30°C) plates. Before recording the contraction assay videos, worms were incubated at RT for 4 min. For further analysis of the loss of contraction during long-term stimulation in SV recycling mutants, the body length was additionally normalized to the time period of 6–10 s during stimulation, and the data was compared during seconds 5–60 of the illumination period. Mean contraction was filtered with a sliding average window of 3 s, and then a linear fit with a forced interception at *y* = 1 was performed, to allow comparing the slope of the loss of contractions (if any).

For analyzing swimming behavior, worms were placed into 96-well plates containing 100 ml NGM and 100 ml of M9 buffer per well. Worms were left in the M9 buffer for 15 min before the first trial of the swimming-assay, then they were filmed for 60 s (swimming-assay before light stimulation). The second round (60 s) of the swimming-assay was filmed after a 90 s light-stimulation and an additional 90 s incubation time. The swimming cycles (the worm’s body bends twice per cycle), were counted manually. The swimming assays were repeated on three different days, with 8–10 worms/group, picked from different populations on different days.

### Aldicarb Assay

Worms were cultivated at 20°C on NGM dishes with OP50 bacteria. To study aldicarb sensitivity, 1.5 mM aldicarb dishes were prepared (Mahoney et al., 2006). After transferring the animals (15–20 young adults/trial, in total three trials on three different days, with worms picked from different populations) to the dishes, they were scored every 30 min by three gentle touches with a hair pick. The assays were performed blinded and on the same day with the same batch of aldicarb dishes.

### Electrophysiology

For recordings from BWMs animals were immobilized with Histoacryl glue (B. Braun Surgical, Spain) and a lateral incision was made to access neuromuscular junctions (NMJs) along the ventral nerve cord. The basement membrane overlying muscles was enzymatically removed by incubation in 0.5 mg/ml collagenase for 10 s (C5138, Sigma-Aldrich, Germany). Muscles were patch-clamped in whole-cell mode at 22°C using an EPC10 amplifier with head stage connected to a standard HEKA pipette holder for fire-polished borosilicate pipettes (1B100F-4, Worcester Polytechnic Institute, Worcester, MA, USA) of 4–7 MΩ resistance. The bath solution contained: NaCl 150 mM; KCl 5 mM; CaCl_2_ 5 mM; MgCl_2_ 1 mM; glucose 10 mM; sucrose 5 mM; HEPES 15 mM, pH 7.3 with NaOH, ~330 mOsm. The pipette solution contained potassium-gluconate 115 mM; KCl 25 mM; CaCl_2_ 0.1 mM; MgCl_2_ 5 mM; BAPTA 1 mM; Na_2_ATP 5 mM; Na_2_GTP 0.5 mM; cAMP 0.5 mM; cGMP 0.5 mM; HEPES 10 mM, pH 7.2 with 1 M KOH, ~320 mOsm. Recordings were conducted at a holding potential of −60 mV. Light activation was performed using a LED lamp at 470 nm (KSL-70, Rapp OptoElectronic, Germany; 8 mW/mm^2^) and controlled by Patchmaster software (HEKA, Germany). Data were analyzed by Patchmaster (HEKA, Germany) and MiniAnalysis (Synaptosoft, USA) software.

### Electron Microscopy

Transgenic L4 worms were transferred from regular NGM dishes to freshly seeded *E. coli* OP50 −/+ (0.1 mM) ATR dishes 1 to 2 days before high-pressure freezing (HPF). Young adult animals were used for HPF fixation, based on methods previously described (Rostaing et al., 2004; Weimer, 2006; Kittelmann et al., 2013). Briefly, about 10–40 worms were loaded into a 100 μm deep aluminum planchette (Microscopy Services) filled with *E. coli* OP50 −/+ ATR, covered with a 0.16 mm sapphire disc and a 0.4 mm spacer ring (Engineering office M. Wohlwend GmbH) for subsequent photostimulation. To prevent preactivation, all manipulations were done under red light. For light stimulation experiments, worms were continuously illuminated with a laser (~20 mW/mm^2^) for 30 s, followed by HPF at −180°C under 2100 bar pressure in a Bal-Tec HPM010 HPF machine. A ~6 s period of manual insertion of the sample after photostimulation is required with this high pressure freezer. For experiments involving the clathrin mutant, animals were kept at permissive temperature (15°C) at all times. Tools used were equilibrated to the respective temperatures. Worms were either loaded at room temperature to the planchette for freezing directly or incubated at non-permissive temperature at 30°C for 14 min before freezing.

After freezing, specimens were transferred under liquid nitrogen into a Reichert AFS machine (Leica) for freeze substitution. Tannic acid (0.1% in dry acetone) fixative was used to incubate samples at −90°C for 100 h. Then, a process of washing was performed, followed by an incubation of 2% OsO_4_ for 39.5 h (in dry acetone) while slowly increasing the temperature up to room temperature. Afterwards, the process of embedding in Epoxy resin (Agar Scientific, AGAR 100 Premix kit hard) was executed with increasing concentration from 50% to 100% at room temperature and 100% at 60°C over 48 h.

For electron micrographs, cross sections were cut at a thickness of 40 nm, transferred to formvar-covered copper slot grids and counterstained in 2.5% aqueous uranyl acetate for 4 min, followed by washing with distilled water. Then, grids were carried onto Reynolds lead citrate solution for 2 min in a CO_2_-free chamber and subsequently washed in distilled water again. Images of regions in the ventral nerve cord were taken with an Erlangshen ES500W CCD camera (Gatan) in a Philips CM12 transmission electron microscope operated at 80 kV. Images were scored blind for each condition and tagged in ImageJ (NIH) for the PM (synapse perimeter). ImageJ ROIs were stored and then quantified based on methods described previously (Steuer Costa et al., 2017). The diameters of synapses from each stimulation condition vary due to the different extent of SV exocytosis or because different synapses were sampled. Thus, each value for the number of docked SVs was normalized and represents the number of docked SVs along a membrane whose perimeter is 1548 nm in a profile; the other organelles are represented as the numbers of SVs or LVs in a typical synaptic profile of 164,100 nm^2^ (Steuer Costa et al., 2017). SV size was scored from 3 to 10 middle images of randomly selected synapses or from randomly selected single images per synapse for each mutant or stimulation protocol. Details (2–3 worms, and typically 10–19 synapses were analyzed per genotype and condition; 1–2 technical replicates were performed) are given in the respective figure legends, and in Supplementary Table S1.

### Bioinformatics

Clustal X2 (Larkin et al., 2007) was used for alignment of endophilin sequences. Alignments were colored using *Boxshade*^1^. The phylogenetic tree was drawn using *Phylodendron*^2^.

### Statistics

The respective statistics used are indicated for each experiment in the figure legends.

## Results

### Endophilin B Is Encoded by Endophilin-Related Protein in *C. elegans*

In *C. elegans*, endophilin A and B are encoded by *unc-57* and *erp-1*, respectively. They show 38.7% (UNC-57 isoform a) and 40.2% (ERP-1 isoform a) identity to their human homologs (Supplementary Figure S1). While the role of UNC-57 in SV recycling was studied earlier and its main sites of action appear to be endocytosis as well as the budding of new SVs from the synaptic endosome (Schuske et al., 2003; Kittelmann et al., 2013), the role of ERP-1 is less clear (Wabnig et al., 2015). For example, could ERP-1 be required for formation of bulk endocytosis structures (Figure 1)? We had previously observed in optogenetic stimulation experiments that *erp-1* mutants showed a slight reduction in photoevoked ePSCs (Wabnig et al., 2015). However, no long-term behavioral assays were performed, to assess SV recycling defects under more robust neuronal stimulation conditions. Also, no detailed expression pattern analysis of *erp-1* in motor neurons was reported so far, and we wanted to confirm that phenotypes of *erp-1* mutants on cholinergic transmission are caused directly in the cholinergic cells and not indirectly in other tissues missing ERP-1.

### ERP-1 Is Expressed in Cholinergic Motor Neurons

**Figure 2:**
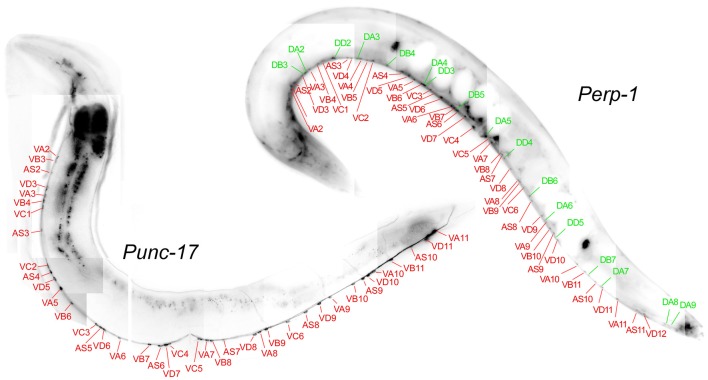
ERP-1 is expressed in cholinergic and GABAergic motor neurons. Fluorescent micrographs, assembled from smaller images, of each one representative animal expressing GFP from the *erp-1* promoter (top right) or channelrhodopsin-2 (ChR2)::YFP (i.e., associated with membranes) from the *unc-17* promoter (bottom left; specific for cholinergic neurons). Tentative assignment of the ventral nerve cord neuronal cell bodies (starting from the midbody region, i.e., the vulva) shows that ERP-1 is expressed in cholinergic (red lines and neuron names) and GABAergic (green lines and names) neurons, while p*unc-17* is restricted to the cholinergic neurons only. See Supplementary Figure S2 for a close-up of the vulva region.

We used an *erp-1* promoter GFP transcriptional fusion and compared the expression pattern to that of p*unc-17*::ChR2::YFP as a marker for cholinergic cells (Figure 2). GFP expression was visible in many tissues, as reported in an earlier high-throughput study (Hunt-Newbury et al., 2007); we focused on the ventral nerve cord motor neuron cell bodies. This part of the nervous system contains cholinergic and GABAergic motor neurons, that can be identified based on anatomical features (position relative to the vulva, left or right commissural processes to the dorsal side, etc.). When compared to the p*unc-17* reporter, it was obvious that ERP-1 is expressed in many more neurons in the ventral nerve cord than just the cholinergic motor neurons (i.e., in >63 visible neurons, vs. only 43 neurons in the p*unc-17* reporter strain). This suggests that ERP-1 is present in GABAergic neurons as well (as these are the only other cell type present in this part of the nervous system). We tentatively assigned the individual cell bodies (Figure 2; Supplementary Figure S2), verifying this notion. Thus, the lack of *erp-1* should lead to direct effects in cholinergic cells, particularly in single-cell assays, such as in electrophysiological recordings and EM analyses.

### Animals Lacking ERP-1 Are Mildly Affected for Cholinergic Transmission

**Figure 3:**
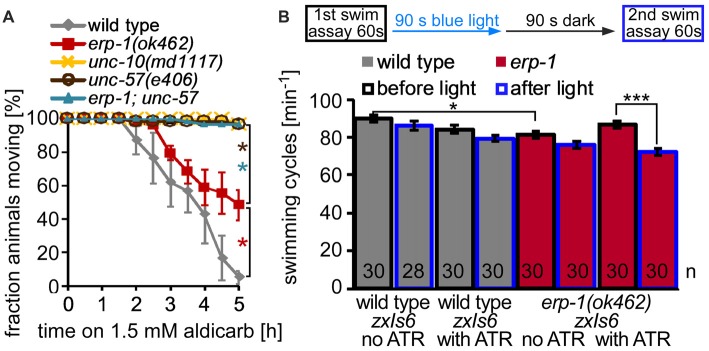
Mutants lacking ERP-1 have mild cholinergic phenotypes. **(A)** Aldicarb assay, showing fraction of non-paralyzed animals after the indicated time on 1.5 mM aldicarb. Wild-type animals were compared to *erp-1(ok462)*, *unc-57(e406)* and *erp-1; unc-57* double mutants. *unc-10*/RIM mutants are used as positive controls. For each assay, three experiments with 15–20 animals from different populations were pooled. Shown are means and SEM, statistically significant differences were analyzed for each time point by *t*-test (*p < 0.05). **(B)** Swimming cycles per minute were counted for wild-type and *erp-1* mutant animals (from three different populations, on three different days) and averaged. Top: scheme of the experiment. A first swimming assay was followed by 90 s of blue light stimulation, a 90 s resting period in the dark, and a second swimming assay. Animals of the indicated genotype (*n* = 28–30 each), all expressing ChR2 in cholinergic neurons (transgene *zxIs6*), were either raised in the absence or presence of the essential ChR2 co-factor all-trans retinal (ATR). Shown are means and SEM. ***p < 0.001, *p < 0.05, after paired or unpaired *t*-test, with Bonferroni correction.

To probe for ERP-1 function in cholinergic cells, we first performed a simple pharmacological assay for malfunction of cholinergic transmission. Aldicarb is an ACh esterase inhibitor that leads to accumulation of ACh in the synaptic cleft and to progressive paralysis of the animals. In mutants with reduced cholinergic transmission, paralysis is delayed or does not occur (Miller et al., 1996). Mutants lacking *erp-1* showed a mild *ric* (resistant to inhibitors of choline esterase) phenotype, while mutants in *unc-57*, and *erp-1; unc-57* double mutants showed very strong *ric* phenotypes and did not paralyze at all during 5 h of exposure to aldicarb (Figure 3A), just as the positive control *unc-10* (encoding rab3-interacting molecule—RIM). This demonstrates that *erp-1* mutants are somewhat affected for cholinergic transmission, however, much less than *unc-57* endophilin A mutants.

### Long-Term Activity Exhausts Synapses Deficient in *erp-1*

Another behavior that can report on cholinergic function at the NMJ is the swimming locomotion of *C. elegans*. Nematodes exhibit rapid swimming behavior in liquid, and bend their body up to 110 times min^−1^, while cholinergic mutants show lower swimming rate or are not swimming at all, depending on the severity of the mutation. Under standard conditions, mutants lacking *erp-1* showed a slight, but significant reduction of swimming locomotion compared to wild-type animals (Figure 3B). We wondered, if under conditions of increased cholinergic transmission, a possible SV recycling defect may affect swimming more pronouncedly. Thus, we used animals expressing channelrhodopsin-2 (ChR2) in cholinergic cells (transgene *zxIs6*, Liewald et al., 2008), and assessed them in swimming assays before 90 s continuous photostimulation of the cholinergic neurons, as well as after a recovery period of 90 s in a second swimming assay. While wild-type animals did not show a significant reduction of swimming rate in the second assay, regardless if the animals were cultivated in the presence or absence of the ChR2 co-factor ATR; (i.e., leading to functional or non-functional ChR2, respectively), *erp-1* mutants raised with ATR showed significantly less swimming cycles after the light stimulus. Thus, *erp-1* mutants show fatigue, likely due to reduced cholinergic transmission, upon light-evoked “exercise”.

**Figure 4:**
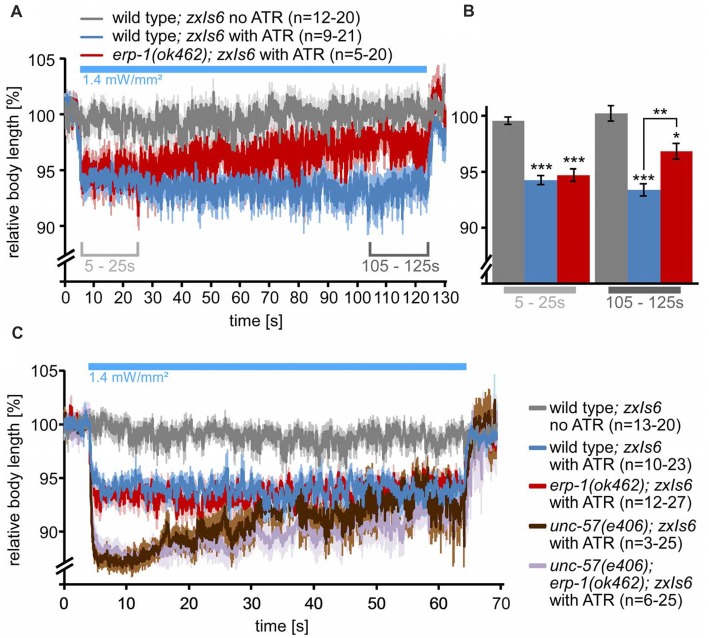
Long-term photostimulation of cholinergic neurons uncovers a behavioral correlate of SV recycling defects in *erp-1* and *erp-1; unc-57* double mutants. **(A)** Contraction assay. Indicated numbers of animals of the indicated genotypes and ATR conditions were photostimulated for 2 min and filmed. Automated analysis of the body length demonstrates sustained contraction in wild-type animals, while *erp-1* mutants show a progressive relaxation of the body length. Animals raised without ATR show no contraction. Means (thick lines) and SEM (lighter shades) of the body length of animals that were analyzable for each time point (at 30 frames per second). In some cases, this number was low for single time points (indicated for n numbers; see Supplementary Figures S3B,C for the numbers of analyzable (i.e., non-coiling) animals at each time point during the assay). Statistically significant differences, shown in **(B)** as means of means, were analyzed for the indicated time periods (5–25 s and 105–125 s). Paired and unpaired *t*-tests, with Bonferroni correction. ***p < 0.001; **p < 0.01; *p < 0.05. **(C)** Contraction assay as in **(A)**, but for 1 min only, and comparing wild type, *erp-1(ok462)*, *unc-57(e406)* as well as *unc-57; erp-1* double mutants. For a statistical analysis of data in **(C)**, see Supplementary Figure S3A.

To further examine this, we used an assay that allows to (indirectly) measure the rate of cholinergic transmission in live animals while they are undergoing strong neuronal activity. Here, animals expressing transgene *zxIs6* are assessed for the extent of the evoked muscle contraction by video microscopy. ChR2 photoactivation leads to spastic paralysis due to simultaneous activation of all body wall muscle cells, and animals show a (longitudinal) contraction of the body (Liewald et al., 2008). Animals with SV recycling defects cannot sustain the contraction for prolonged stimulation periods. When we photostimulated wild-type animals, raised in the presence of ATR, for 2 min, they contracted by about 6% of the body length, until the end of the photostimulus (Figures 4A,B). In contrast, *erp-1* mutants initially contracted also by about 6%, but then progressively reduced the contraction to only 3% at the end of the stimulus period (Figures 4A,B). This difference to wild type was statistically significant. Animals raised in the absence of ATR did not show contractions, as expected. Thus, *erp-1* mutants exhibit typical phenotypes of a SV recycling defect, even though this was less prominent than for endophilin *unc-57* mutants, which lost the contraction almost completely already after 30 s of stimulation (our previous report; Kittelmann et al., 2013). We wondered if *erp-1* and *unc-57*, based on this assay, have redundant functions or could be part of independent pathways, which could indicate different sites of action of the two proteins. Thus, we analyzed *erp-1; unc-57* double mutants and compared them to either single mutant (Figure 4C; see Supplementary Figure S3A for statistical analysis, and for analysis of the number of measurable animals during the time course of the assay, when many animals show increased coiling; Liewald et al., 2008). However, the *unc-57* mutation dominated the phenotype, and *erp-1; unc-57* double mutants did not show an exacerbated phenotype; if at all, they even showed a less prominent reduction of the contraction than *unc-57* single mutants (see also Figure 9; Supplementary Figure S6). Possibly, complete absence of endophilin function may have uncovered a “backup” mechanism that allows for SV recycling in the absence of these proteins.

**Figure 5:**
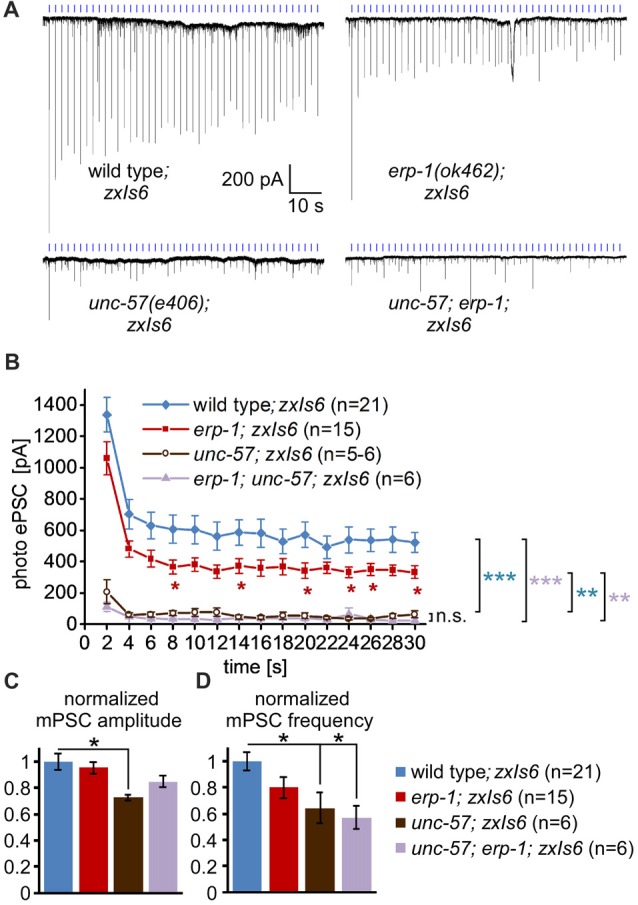
Electrophysiological analysis of spontaneous and photoevoked synaptic transmission in *erp-1* and *unc-57* mutants. **(A)** Representative original records of postsynaptic currents recorded in voltage clamp mode from body wall muscle cells (genotypes as indicated), downstream of photostimulated cholinergic neurons. Light pulses (blue tick marks) were presented at 0.5 Hz. **(B)** Mean and SEM of peak currents evoked by the first 15 photostimuli in the respective animals (numbers are indicated). Statistically significant differences were determined by one-way analysis of variance (ANOVA) with Bonferroni correction; ***p < 0.001; **p < 0.01; *p < 0.05. **(C,D)** Mean, normalized mPSC amplitudes **(C)** and frequencies **(D)** in the indicated animals. One-way ANOVA with Dunnett’s multiple comparisons test; *p < 0.05.

**Figure 6:**
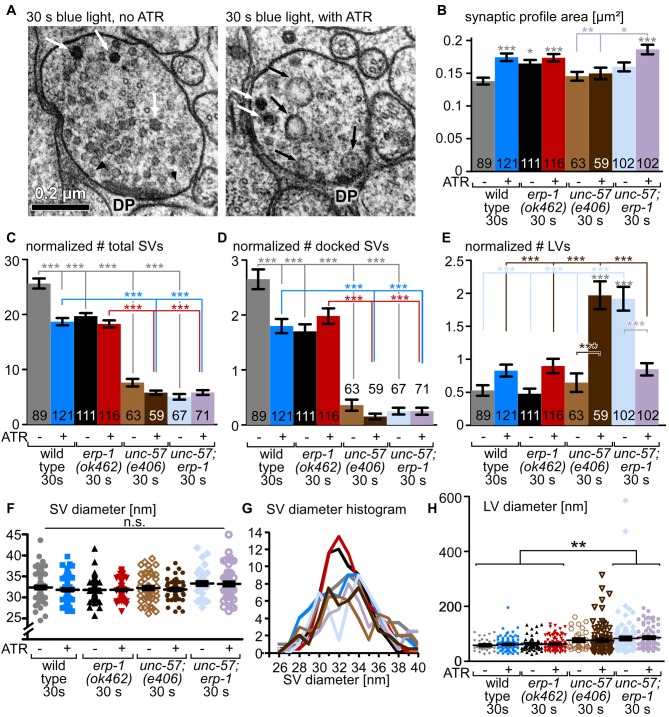
Transmission electron microscopy (EM) analysis of *unc-57*, *erp-1* and *unc-57;* *erp-1* mutants. **(A)** Example thin sections of wild-type cholinergic synapses expressing ChR2 (transgene *zxIs6*, as in all panels in this figure), photostimulated for 30 s, and high-pressure frozen after an additional 5–6 s. Left: animal was raised in the absence of ATR. Right: animal was raised in the presence of ATR. Relevant structures are indicated: DP: proteinaceous material at the center of the active zone; black arrowheads: docked SVs; white arrows: dense core peptidergic vesicles; black arrows: LVs, end-products of ultrafast/bulk endocytosis events. **(B)** Mean and SEM analysis of the dimensions (area) of the synaptic profiles analyzed for each condition and genotype, as indicated. Two to three animals and 9–19 synapses, with 59–121 profiles were analyzed for each condition; the *erp-1; unc-57* double mutant was analyzed in two technical replicates (see Supplementary Table S1). **(C–E)** Mean and SEM analysis of structures (total SVs, docked SVs, LVs, as indicated) counted in the observed synaptic profiles, normalized to a typical synaptic profile area or perimeter (for docked SVs), for the indicated genotypes and experimental conditions. **(F,G)** Analysis of SV diameter (single SVs scatter plot, mean and SEM in **(F)**; occurrence of distinct SV diameters, smoothed with a sliding average of the two neighboring values in the histogram, in **G**); for an analysis of mean SV diameters, see Supplementary Figure S4. **(H)** Analysis of mean and SEM of observed LV diameters, incl. scatter plot of largest diameter of the single LVs observed. Statistical analysis: one-way ANOVA with Tukey correction in **(B–F,H)** ***p < 0.001; **p < 0.01; *p < 0.05.

**Figure 7:**
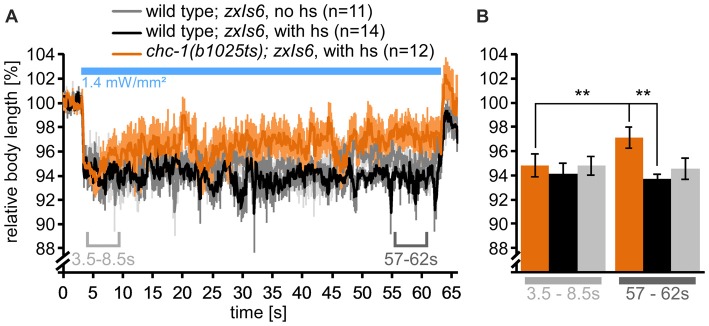
Mutants lacking functional CHC-1 have a mild SV recycling defect. **(A)** Analysis of body length during cholinergic neuron photostimulation, as in Figure 4C, but for wild type or *chc-1(b1025ts)* mutants, cultivated at permissive temperature (15°C), and then shifted (or not) to non-permissive temperature (hs: heat shock; 30°C) for 15 min before the start of the assay. Means (thick lines) and SEM (lighter shades) of the body length. **(B)** Statistical analysis of the time points (means of means) indicated in **(A)**, 3.5–8.5 s and 57–62 s. Paired and unpaired *t*-tests, with Bonferroni correction; **p < 0.01. See also Supplementary Figure S5.

**Figure 8:**
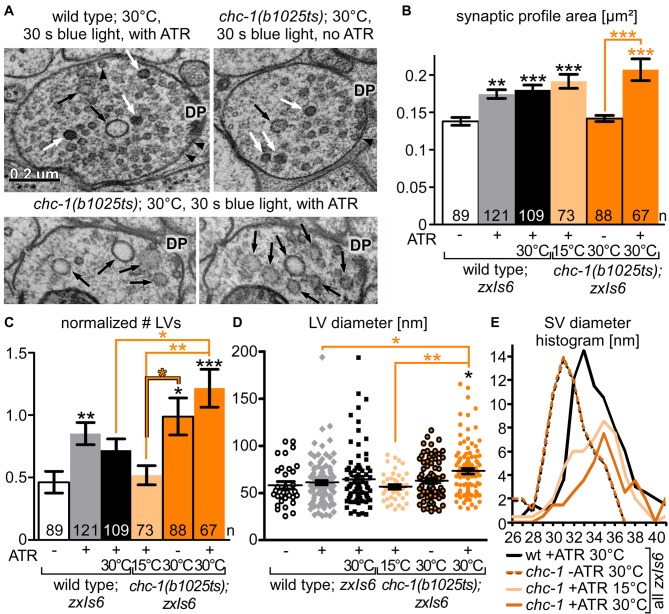
Ultrastructural analysis of *chc-1* mutants demonstrates a role in synaptic endosome breakdown during SV recycling. **(A)** Example electron micrographs of wild-type and *chc-1(b1025ts)* mutant synapses, photostimulated for 30 s under non-permissive temperature (30°C), in animals raised without or with ATR. Structures are indicated as in Figure 6A. **(B)** Synaptic dimension (area) for the indicated genotype and conditions, for the indicated number of profiles, analyzed as in Figure 6B. Two to three animals and 11–19 synapses, with 67–121 profiles were analyzed for each condition (see Supplementary Table S1). **(C)** LV numbers observed per profile, normalized to a typical synaptic profile area, and averaged across the indicated number of profiles. **(D)** Individual LV diameters, shown as scatter plot with means and SEM. Statistical analysis: one-way ANOVA with Tukey correction in **(B–D)**; ***p < 0.001; **p < 0.01; *p < 0.05. **(E)** SV diameter distribution, as a histogram, filtered with a sliding average across the neighboring two values.

**Figure 9:**
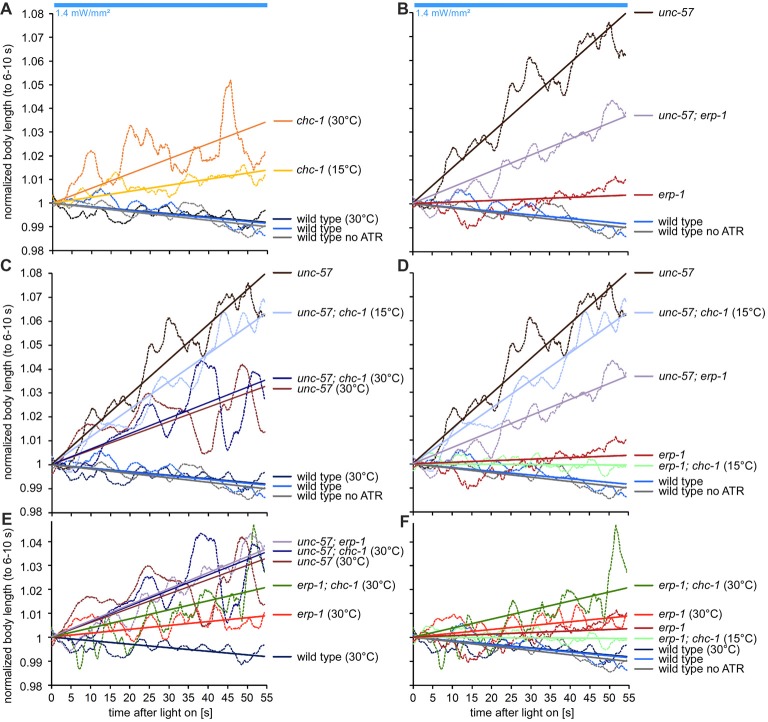
Analysis of evoked contractions in *erp-1;* *chc-1* and *unc-57;* *chc-1* double mutants uncovers mutual rescue of the lack of SV recycling factors. **(A–F)** Contraction analysis of the body length only during the photostimulation, normalized to the contraction during 6–10 s of the stimulus (for graphical explanation of the procedure, as well as number of animals analyzed for each condition, see Supplementary Figure S6). A linear fit was used to compare the slope of the reduction in the contractions. Individual panels show different comparisons (e.g., grouped by mutants used, or hs conditions), for easier comprehension of the data.

### Physiological Phenotypes of Mutants Lacking ERP-1 and UNC-57

To further investigate this, we used electrophysiology to measure spontaneous and optogenetically evoked transmission at the NMJ (excitatory post-synaptic currents—ePSCs). Also these assays can uncover SV recycling defects (Liewald et al., 2008). Optogenetic stimulation of the cholinergic neurons at 0.5 Hz, which evokes strong photo-ePSCs in wild type, caused significantly smaller currents in *erp-1* mutants (*ca*. 2/3 of wild type; Figures 5A,B). In *unc-57* mutants, photo-ePSCs were ~15% of wild-type photo-ePSCs for the first stimulus, and then almost immeasurable for further stimuli. *unc-57; erp-1* double mutants had essentially the same phenotype as *unc-57* single mutants. However, these strong phenotypes were particularly evident for stimulated transmission, while for spontaneous miniature post-synaptic currents (mPSCs), only *unc-57* mutants showed slightly reduced amplitudes. In contrast, both *unc-57* and *unc-57; erp-1* double mutants showed significantly reduced mPSC frequency, as compared to wild type. This is likely a consequence of reduced SV numbers. Thus, endophilin A/UNC-57 appears to have much more pronounced functions in SV recycling and maintenance of synaptic transmission than endophilin B/ERP-1.

### Synaptic Ultrastructure of *erp-1* Mutants Shows a Defect in the Breakdown of Bulk-Endocytosed Large Vesicles (LVs)

To directly analyze the site of action of ERP-1, and the morphology of the synapse under resting and stimulated conditions, we turned to ultrastructural analyses of thin sections of cholinergic *en passant* synapses (flash’n’freeze EM; Kittelmann et al., 2013; Watanabe et al., 2013a). Briefly, animals expressing ChR2 in cholinergic neurons were photostimulated for 30 s with blue light, and then transferred into the freezing chamber of a high pressure freezer (this process requires 5–6 s), which can cryo-fix the tissue within ms. This freezes all morphological changes and features in native conditions. Following a freeze-substitution and heavy metal staining protocol, the synaptic ultrastructure is then analyzed in 40 nm thin sections by transmission EM (TEM; Figure 6A). Photostimulation of *zxIs6* synapses in animals raised in the absence of ATR did not evoke any obvious effects in the synapse, while the stimulation of functional ChR2 (animals raised with ATR) led to drastic alterations in the synaptic profile: first, the synaptic profile area was significantly increased, which we interpret as a result of excessive SV fusion that adds membrane to the PM of the *en passant* synaptic bouton (Figure 6B). Consequently, the total number of SVs was reduced for wild-type animals (Figure 6C), as was the number of docked SVs, i.e., SVs that are in zero visible distance to the PM and thought to be ready for fusion (i.e., primed SVs; Figure 6D). In addition, large, clear-core vesicles (LVs), end-products of bulk/ultrafast endocytosis, were observed at elevated numbers in stimulated synapses (Figure 6E), though this did not meet significance under conditions of multiple-comparisons statistics. We compared wild type to *erp-1* mutants. These had enlarged synaptic profile areas, even without stimulation, possibly due to inefficient SV recycling; however, they exhibited a reduced total number of SVs even before stimulation. This was no further reduced by light stimulation, indicating that the readily releasable pool (RRP) and/or RP are reduced in these animals, and that a lower turnover of SVs occurs, in line with our behavioral and electrophysiological assays. *erp-1* mutants also showed reduced docked SV numbers, that were not further reduced upon photostimulation. LV numbers in *erp-1* were increased upon stimulation, just as for wild type. Next, we analyzed endophilin A/UNC-57 mutants: *unc-57(e406)* synapses had significantly reduced numbers of total and docked SVs (~30% and <15% of wild type, respectively), that were further depleted upon stimulation, indicating a strong defect in SV formation and/or recycling, consistent with the electrophysiological results (Figure 5). Upon stimulation, *unc-57* mutants showed a strong increase (~4 times more than wild type) in the number of LVs per synaptic profile (this was unexpected given our previous findings; Kittelmann et al., 2013), where *unc-57* mutants had less, but larger LVs than wild type; the difference is due to different analyses: previously, we counted LVs per synapse, not per thin section profile; if we do the same here, we see no major difference to wild type, i.e., 2.2 vs. 2 LVs per synapse, respectively. Consistently, LV size was increased in *unc-57* mutants (Figure 6H). Thus, UNC-57/endophilin A is not essential for formation of LV/endosomes upon synaptic stimulation, yet these LVs cannot be broken down into new SVs. ERP-1 also contributes to formation of SVs, as no LVs accumulate in its absence; yet, its site of action remains unclear.

To investigate this, we also analyzed *unc-57; erp-1* double mutants. These animals showed significantly increased synapse cross-sections after 30 s photostimulation (Figure 6B), indicating that some SV fusion took place, despite the very low number of fusion events evident from electrophysiological analysis of these mutants (Figure 5), and that an impairment in endocytosis may occur. Just as *unc-57* mutants, *unc-57; erp-1* double mutants had extremely reduced total SV and docked SV numbers (~20% and ~10% of wild type, respectively). In contrast to wild type and also to *unc-57* single mutants, the double mutants showed highly increased numbers of LVs (~350% of wild type), already before stimulation. This indicates major difficulties of these synapses to break down the end-products of bulk endocytosis; yet, endocytosis *per se* does occur. LVs in *erp-1; unc-57* double mutants were significantly larger than in wild type or *erp-1* single mutants (Figure 6H). After stimulation, the numbers of LVs, surprisingly, were reduced compared to the non-stimulated condition, and were comparable to stimulated wild type or *erp-1* synapses. This was in strong contrast to *unc-57* mutants. This indicates that in the absence of all endophilins, (activity-, possibly Ca^2+^-dependent) mechanisms exist that enable some formation and recycling of LVs, such as to sustain a low level of SV exocytosis and recycling.

We also analyzed the diameter of SVs, to assess whether the regulation of the organelle characteristics is affected. SV diameters did not exhibit any obvious difference in the mean, or in the diameter distribution in wild type, *erp-1* and *unc-57; erp-1* double mutants, neither under resting or stimulation conditions (Figures 6F,G; Supplementary Figure S4).

### Clathrin Mutants Have a SV Recycling Defect

We next asked at which stage during SV recycling clathrin function is required. Clathrin was shown to affect SV size in *C. elegans* (SVs became smaller in the absence of functional clathrin heavy chain CHC-1; Sato et al., 2009), and in mammalian synapses, clathrin was demonstrated to act at the synaptic endosome (Watanabe et al., 2014). As clathrin heavy chain (encoded by *chc-1*) is essential for survival, we could assess only a *chc-1(b1025ts)* temperature sensitive allele: these animals are comparably unaffected at the permissive temperature (15°C), but are deprived of CHC-1 function at elevated temperatures (30°C). First, we performed a time-dependent optogenetic contraction assay, comparing wild type to *chc-1* mutants at elevated temperature. After the shift to 30°C, the animals showed a (mild) decline in contractions over the 1 min photostimulation period, that was significantly different from wild type and from the initial contraction, while wild-type animals showed comparable and consistent contractions both at 15°C (no hs) and at 30°C (with hs; Figures 7A,B, 7A,B, 9A; Supplementary Figure S5). Thus, *chc-1(b1025ts)* mutants showed a mild SV recycling defect at non-permissive temperature.

### Synapses Lacking Functional Clathrin Heavy Chain Exhibit Numerous and Large LVs

Next, we analyzed the synaptic ultrastructure of *chc-1* mutants, at permissive or non-permissive temperatures (Figure 8A). The synaptic profile area of the analyzed synapses, regardless of wild type or *chc-1* mutation, and regardless of permissive or non-permissive temperature, increased significantly during photostimulation (Figure 8B). This indicates that CHC-1 is not required for SV fusion. When we analyzed LV numbers, *chc-1* mutant synapses showed significantly more LVs than wild-type synapses at the non-permissive temperature (Figure 8C). This was already the case without photostimulation. Thus, CHC-1 is required for SV recycling at the stage of the synaptic endosome, and not at the stage of PM-localized bulk endocytosis. Also, normal synaptic activity is sufficient under the non-permissive conditions to cause accumulation of LVs, which obviously cannot be degraded in the absence of functional CHC-1. Last, we also analyzed the LV diameter and found that *chc-1* mutants had significantly larger LVs at the non-permissive temperature (Figure 8D), in line with a function of CHC-1 in the breakdown of LVs to form new SVs. SV diameters were reduced in *chc-1* mutants at non-permissive temperature, without ATR (and thus without functional ChR2), as observed previously (Sato et al., 2009). However, photostimulation induced a shift to larger (wild-type-like) SV diameters in the *chc-1* mutant, both at permissive and non-permissive temperatures. Possibly, under conditions of evoked SV turnover even the compromised CHC-1 can form normal cages and thus generate normal size SVs; alternatively, other proteins like ERP-1 or UNC-57 can compensate for the lack of clathrin activity.

### Synergistic Activities of Endophilins and Clathrin Enable SV Recycling

Despite the lack of endophilins in the *unc-57; erp-1* double mutant, SVs were still present and some endocytosis and SV recycling persisted. As clathrin was shown in rodent neurons to act at the PM at non-physiological temperatures, and as a back-up mechanism when ultrafast endocytosis is non-functional (e.g., in the absence of actin polymerization; Watanabe et al., 2014), we wondered if clathrin may act in a similar manner in *C. elegans*. To address this, we analyzed *erp-1; chc-1* and *unc-57; chc-1* double mutants. First, we assessed the performance of the NMJ in long term stimulation assays and the resulting body contraction. Since the absolute degree of contraction was very different in different mutants and experimental conditions, and the distinctive phenotype uncovering a defect in SV recycling is the gradual loss of the contraction during the stimulation, we turned to comparing the temporal change (i.e., slope) in (normalized) contractions over the 1 min stimulation period (Figure 9; Supplementary Figure S6). The strongest phenotype (highest slope) was apparent in the *unc-57* single mutant. The *unc-57; erp-1* or *unc-57; chc-1(b1025ts)* double mutants (under non-permissive temperature, affecting CHC-1 function), however, somewhat reduced the extent of this phenotype (Figures 9B,C). This could indicate that when all endophilins, or both endophilin A and clathrin are compromised, a compensation by another mechanism (CHC-1/clathrin, UNC-57/endophilin A, or ERP-1/endophilin B, respectively), takes over to enable some endocytosis and SV recycling to occur. In *erp-1; chc-1* double mutants, only comparably mild phenotypes were apparent (Figures 9E,F), emphasizing that UNC-57 is the “main” player in both endocytosis and endosomal SV recycling.

**Figure 10:**
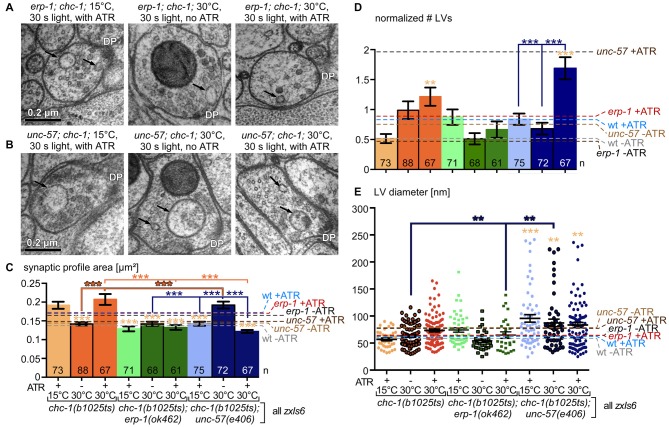
Ultrastructural analysis of *erp-1;* *chc-1* and *unc-57;* chc-1 double mutants confirms mutual rescue of the lack of SV recycling factors. **(A,B)** Example micrographs of *erp-1; chc-1* **(A)** and *unc-57; chc-1* **(B)** mutant synapses, photostimulated for 30 s under permissive (15°C) or non-permissive temperature (30°C), in animals raised without or with ATR, as indicated. Structures are marked as in Figure 6A. **(C)** Statistical analysis of synaptic dimension (area) for the indicated genotype and conditions, for the indicated number of profiles, analyzed as in Figure 6B. Two animals and 9–13 synapses, with 67–88 profiles were analyzed for each condition (see Supplementary Table S1). **(D)** Mean and SEM analysis of number of LVs per profile, normalized to a typical synaptic profile area, for the indicated genotypes and experimental conditions, as indicated. **(E)** Individual LV diameters, shown as scatter plot with means and SEM. Statistical analysis: one-way ANOVA with Tukey correction in **(C–E)**; ***p < 0.001; **p < 0.01. For comparison, in **(C–E)**, data for *chc-1* single mutants is reproduced from Figure 8. Also indicated in **(C–E)** are the respective mean values from other genotypes (Figure 6), as dashed lines and labeled on the right.

To analyze the synaptic correlate of these findings, we analyzed the ultrastructures of the *erp-1; chc-1* and *unc-57; chc-1* double mutants, at permissive and non-permissive temperatures, without and with stimulation (illumination in absence/presence of ATR; Figure 10). In electron micrographs, particularly large and numerous LVs could be observed in *unc-57; chc-1* mutants under hs conditions (Figures 10B,D,E). This was similar for *chc-1* single mutants at the non-permissive temperature (though not as pronounced), as well as for *unc-57* single mutants. However, it was not the case in *erp-1; chc-1* mutants (compare Figures 6E,H, 10A,D,E). Thus, endophilin A, B and clathrin appear to act as mutual back-up mechanisms for SV endocytosis and recycling (with strongest contribution by endophilin A) when the two other players are missing.

## Discussion

Here, we analyzed the role of three proteins in SV recycling at cholinergic NMJs of the nematode *C. elegans*. We found that UNC-57/endophilin A functions at both the PM and at the synaptic endosome, to recycle and generate new SVs. For clathrin, we found that it mainly acts at the endosome in the formation of new SVs, but is not associated with the bulk endocytosis process itself (at the PM), as long as endophilin A is present. Clathrin, however, can compensate for loss of endophilin A also at the PM. ERP-1/endophilin B has only a minor role (its phenotypes were mild, but could be exacerbated by synaptic activity), which can be both at the PM, as suggested by the residual recycling activity that we observed in the *unc-57; chc-1* double mutant, and at the endosome, as suggested by the reduced number of SVs in this mutant. For clathrin, we found that, as in mammals, its function is also associated with the breakdown of synaptic endosomes under physiological conditions. In sum, we could clarify roles for endophilins A, B and clathrin in the SV recycling process in *C. elegans*, uncovering a capacity of each protein for mutual “rescue” of the lack of other two proteins in the different stages of SV recycling.

The role of endophilin A has been controversially discussed. Based on the finding that (very large) bulk endosomes are formed in *unc-57* mutants (this work and Kittelmann et al., 2013), as well as in *unc-57; erp-1* double mutants lacking all endophilins (this work), we suggest that UNC-57 is not absolutely essential for the PM-localized bulk endocytosis event, and that clathrin acts in a back-up pathway that helps in formation of endocytic structures at the PM. This mechanism may be augmented by high Ca^2+^, as we found that numbers of LVs were normal in photostimulated *unc-57; erp-1* double mutants, while these synapses accumulated numerous and large LVs without stimulation. Likewise, a dependency of ADBE on Ca^2+^ and calcineurin was reported earlier (Clayton et al., 2009; Wu et al., 2014). The numbers of LVs were increased in *unc-57* mutants, as well as their diameter. This is in apparent contrast to our earlier work, where the numbers of LVs in *unc-57* mutants (Kittelmann et al., 2013) were smaller than in wild type. Yet, this is due to the different ways of analysis, as we previously counted LVs per entire synapse, while here we counted LV sections per synaptic profile.

In *erp-1* mutants, LVs are formed, likely excluding a primary role for ERP-1 at the PM. Yet, the amount of LVs formed is not increased when compared to wild type. Nonetheless, since *erp-1* mutants have significantly reduced SV numbers, the bottleneck appears to be the resolution of the endosomes to form new SVs. Here, however, also UNC-57 must act, as otherwise we would expect stronger phenotypes in *erp-1* mutants. The lower severity of the *erp-1* mutant vs. the *unc-57* mutant could indicate that *unc-57* phenotypes are a consequence of its lack at both the PM and endosomal membrane, for which ERP-1 cannot compensate, or only when also clathrin is absent. The lack of endophilin A function may have much more severe consequences (most clearly demonstrated by the almost complete lack of evoked activity in our electrophysiological experiments); this may not only be because of its role at the PM and endosome in SV recycling, but likely also because it is required for processes involved in synapse formation. Cargo arriving from the soma, as part of transport vesicles, is thought to be first delivered to the PM by fusion of these vesicles (Hannah et al., 1999). From there, canonical endocytosis is required to retrieve this cargo and sort it to its final destination; for such processes, endophilin A (and synaptojanin) are likely to be required. Also, recycling factors required at the PM, or to cycle between PM and endosome, may become trapped and unavailable for later rounds of recycling. Thus, endophilin A mutants may not only be compromised for SV recycling during acute phases of activity, but also for formation of normal synapses.

Not unexpectedly, the role of clathrin appears highly conserved between lower and higher animals also in the SV recycling process (Watanabe et al., 2014). We did not observe any evidence of CME being required for SV recycling/ultrafast endocytosis, unless endophilin function was compromised. Rather, clathrin acted at the endosome-SV transition: LV numbers were strongly increased in stimulated *chc-1* mutant synapses at the non-permissive temperature, and the size of these LVs was larger than in wild type. This indicates that endosomes accumulate and grow, probably by fusion of multiple LVs, because clathrin-mediated formation of new SVs is blocked. The endosomal site of action puts clathrin in the expected position to control the size of newly formed SVs, and earlier work had demonstrated that SV size is reduced in clathrin mutants (Sato et al., 2009). Also in our hands SVs were smaller in *chc-1* mutants at non-permissive temperature. However, SV size appeared to be normal if the same animals were photostimulated. Possibly, an activity-(Ca^2+^) dependent process that is triggered by ChR2 stimulation could have augmented the compromised CHC-1 protein in forming normal size SVs. This will require future investigation.

In sum, we combined optogenetic, behavioral, electrophysiological and ultrastructural analyses in wild-type and mutant *C. elegans* to assess the roles of endophilin A, B and of clathrin in SV recycling, showing a mutual compensation for the lack of the respective other two proteins. The fact that nematodes can live despite carrying severe mutations, combined with the fact that many genes are present only in single copy in *C. elegans* (e.g., one endophilin A, compared to three isoforms in mammals) facilitate such studies. However, details of the function of these proteins may be different between the species, thus comparisons across different experimental and organismal preparations will remain an important approach.

## Author Contributions

AG: acquired funding and wrote the manuscript, with the help of the other authors. S-cY, BJ, SW, JL and AG: conceived research and performed analyses. S-cY, BJ, SW and JL: performed experiments.

## Conflict of Interest Statement

The authors declare that the research was conducted in the absence of any commercial or financial relationships that could be construed as a potential conflict of interest.

## Footnotes

^1^http://www.ch.embnet.org/software/BOX_form.html

^2^http://iubio.bio.indiana.edu/soft/molbio/java/apps/trees/

## Supplementary Material

FIGURE S1 Homology analysis among human and *C. elegans* endophilins. **(A)** Clustal X2 alignment of human endophilins A1 and B1, as well as *C. elegans* UNC-57 and ERP-1. Asterisks or dots indicate identity in all, or homology in 50% of the sequences, respectively. **(B)** Phylogenetic tree.

FIGURE S2 Neuronal expression patterns of the erp-1 and the unc-17 promoters. Shown is a close-up of the mid-body region of two individual animals in Figure 2, aligned at the vulva structure (flanked by the VC4 and VC5 neurons). Cholinergic neuron cell bodies (red neuron names), tentatively assigned based on their reported positions along the ventral cord, as well as of interspersed GABAergic neurons (green neuron names) are indicated.

FIGURE S3 Statistical analysis of channelrhodopsin-2 (ChR2)-evoked contraction in different mutants, as in Figure 4. **(A)** Mean contraction was compared between seconds 5–10 and 60–65 of the experiment shown in Figure 4C. Statistical analysis: Two-way or one-way *t*-test with Bonferroni correction; ****p* < 0.001; **p* < 0.05. **(B,C)** Numbers of animals that were analyzable (i.e., that were not coiling and could thus be measured faithfully for body length by the video analysis tool) during the time course of the experiments shown in Figures 4A,C.

FIGURE S4 Synaptic vesicle diameters of all genotypes analyzed by transmission electron microscopy (TEM). Shown are the mean and SEM of measured synaptic vesicle (SV) diameters, for the indicated number of SVs. Genotype and experimental conditions are noted under each bar. Statistical analysis: one-way ANOVA with Tukey correction; ****p* < 0.001; ***p* < 0.01.

FIGURE S5 Statistical analysis of non-coiling animals during ChR2-evoked contraction in chc-1 mutants, as in Figure 7. Number of animals that was analyzable during the timecourse of the experiments shown in Figure 7A. hs, heat shock.

FIGURE S6 Comparison of the rate of loss of body contraction during long-term photo-stimulation of cholinergic neurons, as shown in Figure 9. **(A)** Processing behavioral data for a comparison of the slope of the loss of contractions during the photo-stimulation period. Left panel: original data (mean body length) is first normalized to the time window 6–10 s (red bar); normalized data is shown in the middle panel. This data is then filtered with a sliding average and plotted in the right panel. The smoothed mean data during seconds 5–60 of the stimulation period (blue shaded box) is then used for generating linear fits with a forced interception at *y* = 1, as shown in **(B)** and in Figure 9. **(C)** Number of animals that was analyzable during the time course of the experiments shown in Figure 9.

TABLE S1 Statistics of biological and experimental replicates, synapse number and profile number in electron microscopy (EM) analyses of this work. Statistical analysis: one-way ANOVA with Tukey correction; ****p* < 0.001; ***p* < 0.01; **p* < 0.05.
