# Supplementary figures and images for: Endophilin A and B Join Forces With Clathrin to Mediate Synaptic Vesicle Recycling in Caenorhabditis elegans
Source: Front Mol Neurosci. 2018 Jun 14;11:196. doi: 10.3389/fnmol.2018.00196 (PMC6010539; doi:10.3389/fnmol.2018.00196)

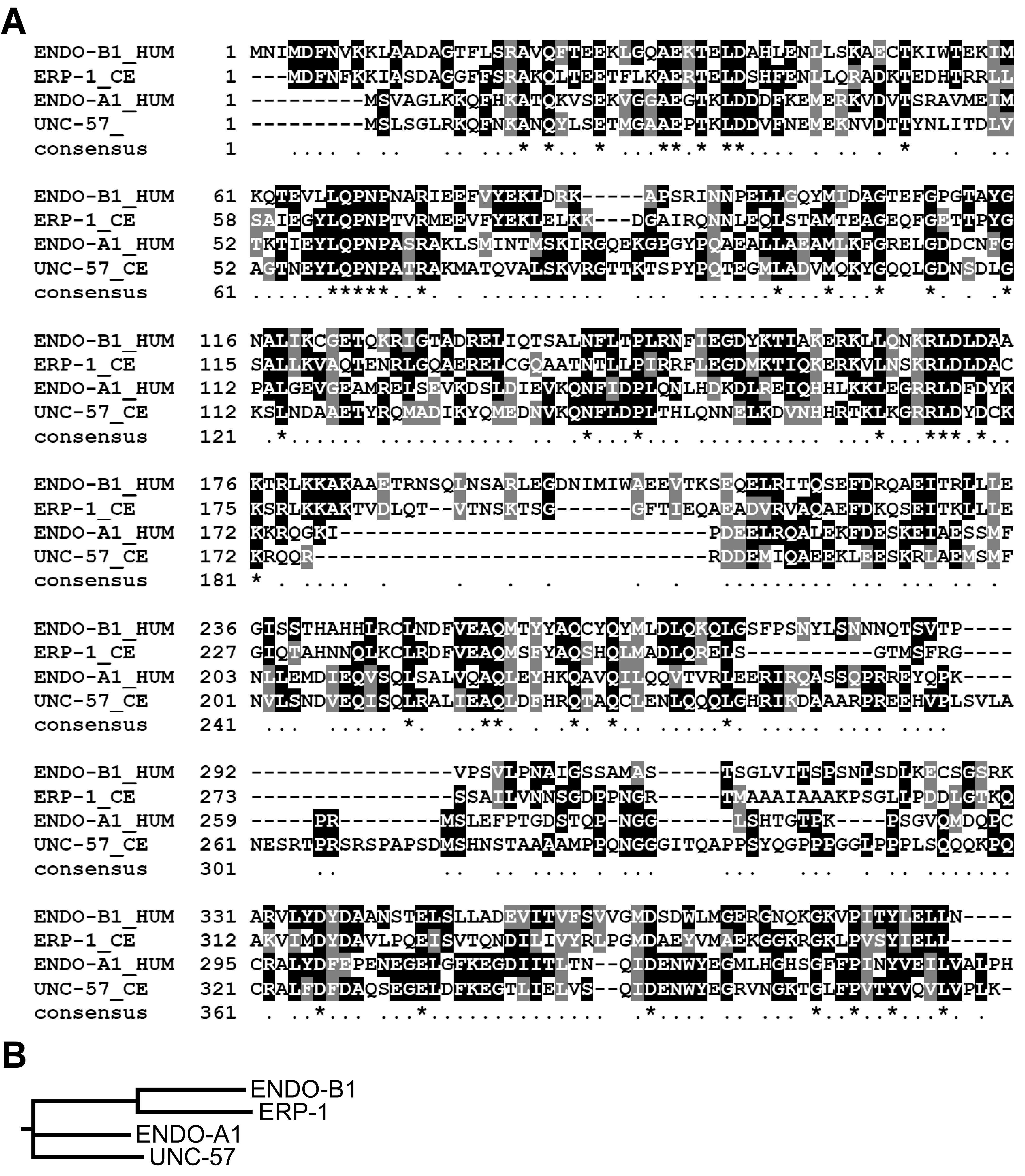

Supplement: FIGURE S1 — Homology analysis among human and C. elegans endophilins. (A) Clustal X2 alignment of human endophilins A1 and B1, as well as C. elegans UNC-57 and ERP-1. Asterisks or dots indicate identity in all, or homology in 50% of the sequences, respectively. (B) Phylogenetic tree. [file Image_1.TIF]

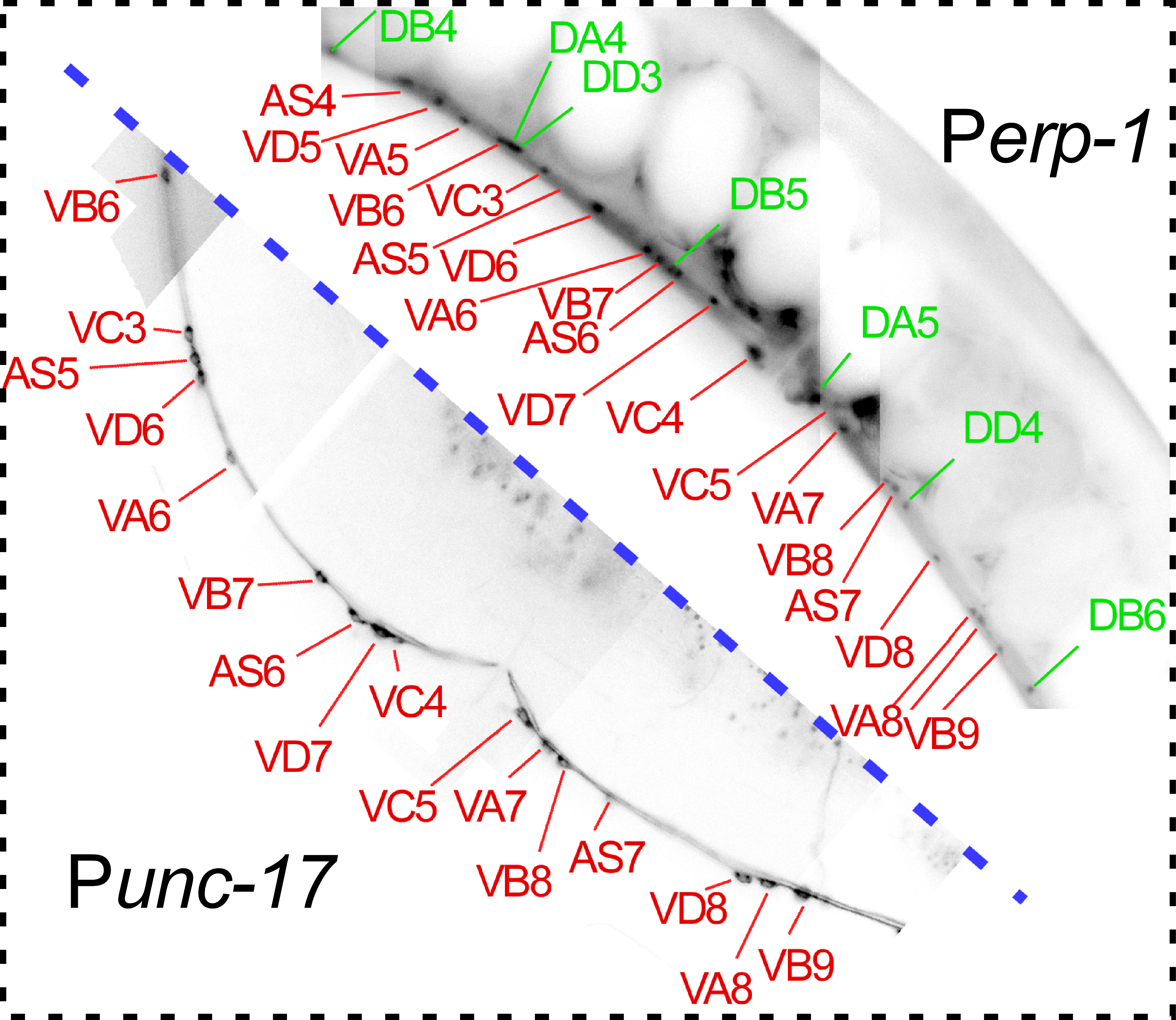

Supplement: FIGURE S2 — Neuronal expression patterns of the erp-1 and the unc-17 promoters. Shown is a close-up of the mid-body region of two individual animals in Figure 2, aligned at the vulva structure (flanked by the VC4 and VC5 neurons). Cholinergic neuron cell bodies (red neuron names), tentatively assigned based on their reported positions along the ventral cord, as well as of interspersed GABAergic neurons (green neuron names) are indicated. [file Image_2.TIF]

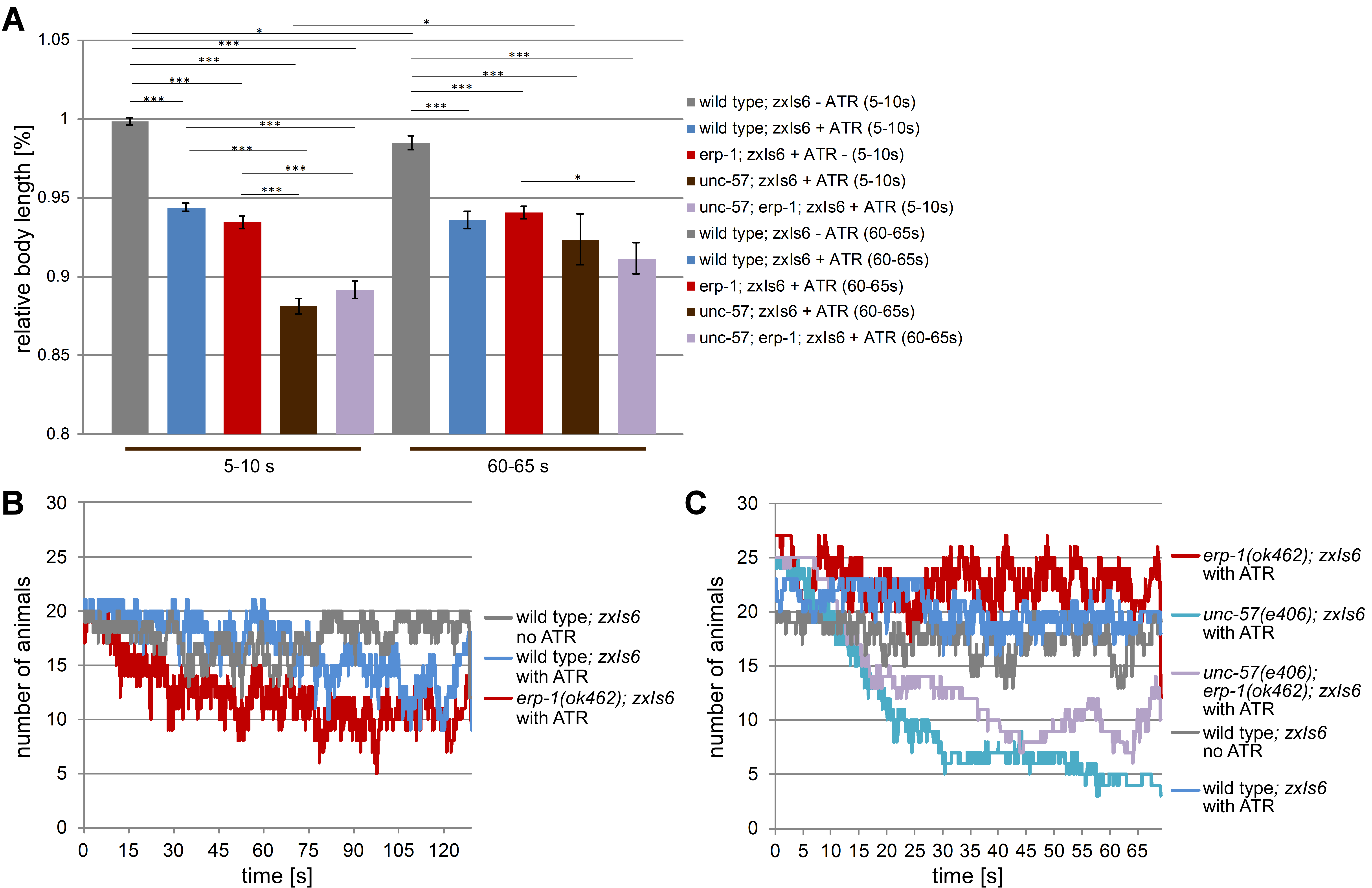

Supplement: FIGURE S3 — Statistical analysis of channelrhodopsin-2 (ChR2)-evoked contraction in different mutants, as in Figure 4. (A) Mean contraction was compared between seconds 5–10 and 60–65 of the experiment shown in Figure 4C. Statistical analysis: Two-way or one-way t-test with Bonferroni correction; ***p < 0.001; *p < 0.05. (B,C) Numbers of animals that were analyzable (i.e., that were not coiling and could thus be measured faithfully for body length by the video analysis tool) during the time course of the experiments shown in Figures 4A,C. [file Image_3.TIF]

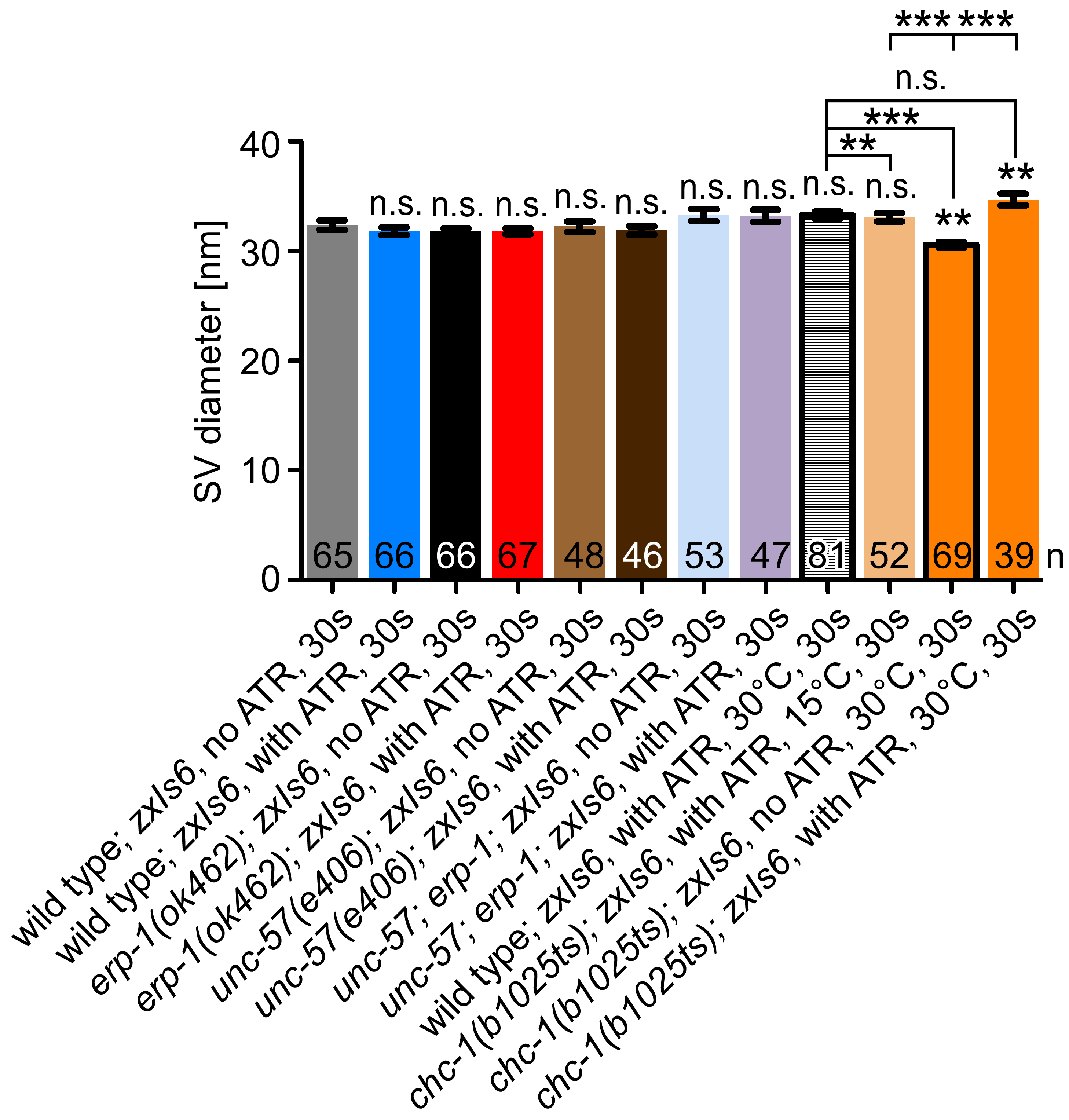

Supplement: FIGURE S4 — Synaptic vesicle diameters of all genotypes analyzed by transmission electron microscopy (TEM). Shown are the mean and SEM of measured synaptic vesicle (SV) diameters, for the indicated number of SVs. Genotype and experimental conditions are noted under each bar. Statistical analysis: one-way ANOVA with Tukey correction; ***p < 0.001; **p < 0.01. [file Image_4.TIF]

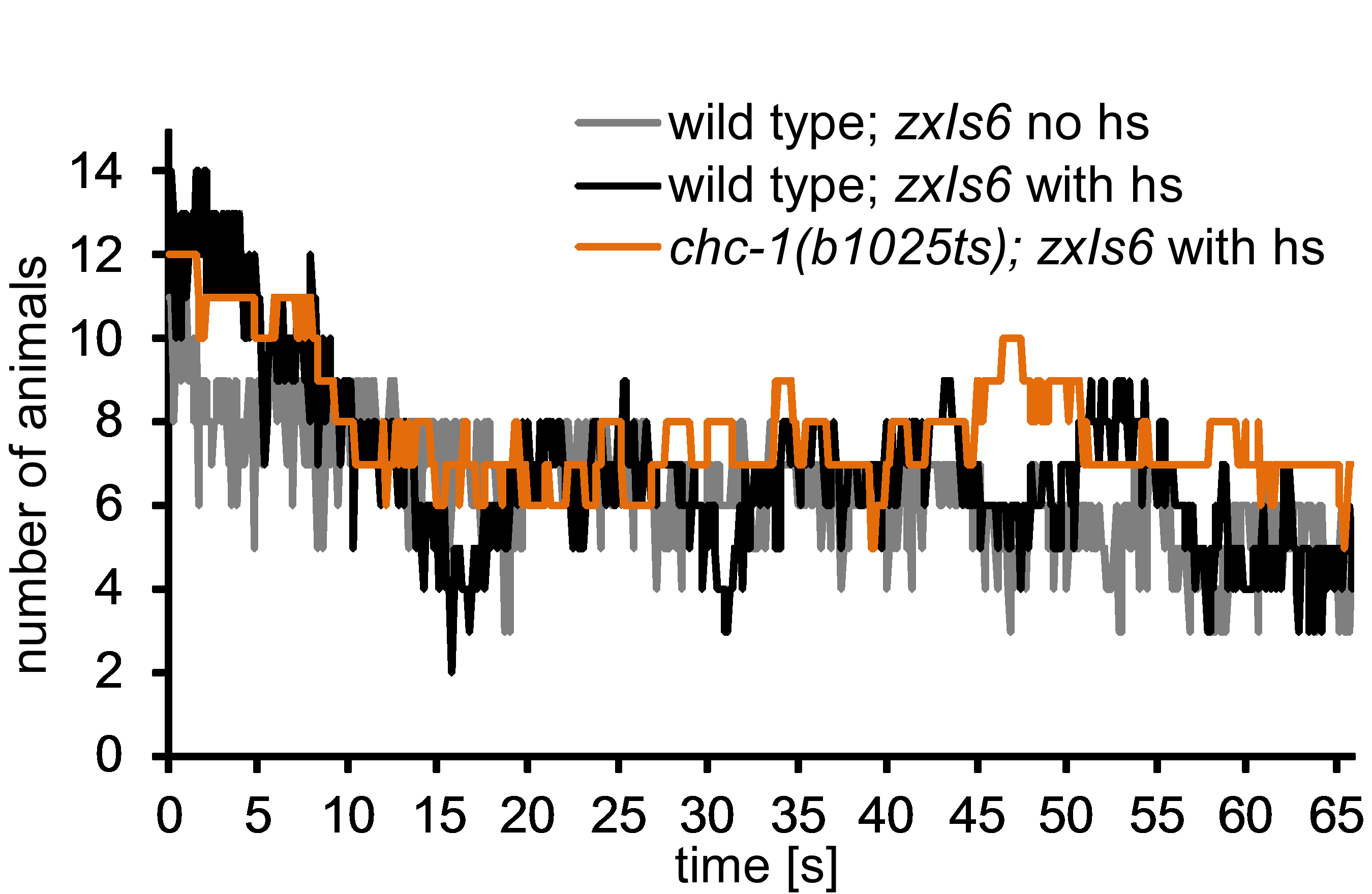

Supplement: FIGURE S5 — Statistical analysis of non-coiling animals during ChR2-evoked contraction in chc-1 mutants, as in Figure 7. Number of animals that was analyzable during the timecourse of the experiments shown in Figure 7A. hs, heat shock. [file Image_5.TIF]

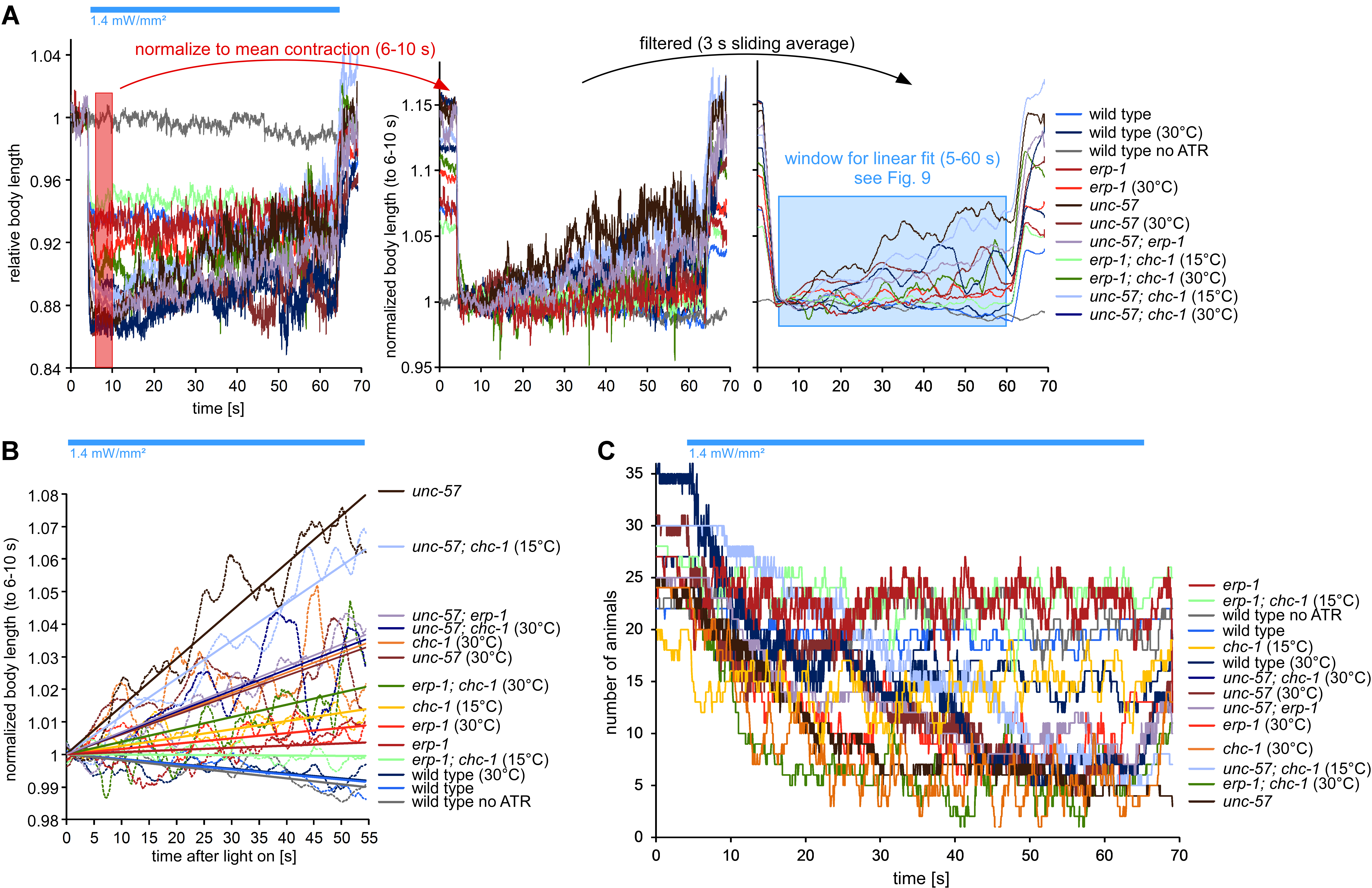

Supplement: FIGURE S6 — Comparison of the rate of loss of body contraction during long-term photo-stimulation of cholinergic neurons, as shown in Figure 9. (A) Processing behavioral data for a comparison of the slope of the loss of contractions during the photo-stimulation period. Left panel: original data (mean body length) is first normalized to the time window 6–10 s (red bar); normalized data is shown in the middle panel. This data is then filtered with a sliding average and plotted in the right panel. The smoothed mean data during seconds 5–60 of the stimulation period (blue shaded box) is then used for generating linear fits with a forced interception at y = 1, as shown in (B) and in Figure 9. (C) Number of animals that was analyzable during the time course of the experiments shown in Figure 9. [file Image_6.TIF]
