# Supplementary material for: Endophilin A and B Join Forces With Clathrin to Mediate Synaptic Vesicle Recycling in Caenorhabditis elegans
Source: Front Mol Neurosci. 2018 Jun 14;11:196. doi: 10.3389/fnmol.2018.00196 (PMC6010539; doi:10.3389/fnmol.2018.00196)
Supplement: TABLE S1 — Statistics of biological and experimental replicates, synapse number and profile number in electron microscopy (EM) analyses of this work. Statistical analysis: one-way ANOVA with Tukey correction; ***p < 0.001; **p < 0.01; *p < 0.05. [file Table_1.docx]

Supplementary Table 1:

| **Figure 6** | one way ANOVA | Tukey correction | |  | *, p≤0.05 | **, p≤0.01 | ***, p≤0.001 |  | all stimulated with 30s blue light | | | |  |  |  |  |  |  |
| --- | --- | --- | --- | --- | --- | --- | --- | --- | --- | --- | --- | --- | --- | --- | --- | --- | --- | --- |
|  |  |  |  |  |  |  |  |  |  |  |  |  |  |  |  |  |  |  |
|  | **WT;zxIs6 -ATR** | | **WT;zxIs6 +ATR** | | **erp-1;zxIs6 -ATR** | | **erp-1;zxIs6 +ATR** | | **unc-57;zxIs6 -ATR** | | **unc-57;zxIs6 +ATR** | | **unc-57;erp-1;zxIs6 -ATR** | | **unc-57;erp-1;zxIs6 +ATR** | |  |  |
| N (synaptic profiles) | 89 | | 121 | | 111 | | 116 | | 63 | | 59 | | 67 or 102, two techn. replic. | | 71 or 102, two techn. replic. | |  |  |
| number of worms analyzed | 3 | | 2 | | 3 | | 2 | | 2 | | 2 | | min 3 | | min 2 | |  |  |
| number of synapses analyzed | 15 | | 19 | | 19 | | 18 | | 9 | | 10 | | min 13 | | min 16 | |  |  |
|  | **mean** | **SEM** | **mean** | **SEM** | **mean** | **SEM** | **mean** | **SEM** | **mean** | **SEM** | **mean** | **SEM** | **mean** | **SEM** | **mean** | **SEM** |  |  |
| area | 0,14 | 0,005 | 0,17 | 0,006 | 0,16 | 0,006 | 0,17 | 0,006 | 0,15 | 0,007 | 0,15 | 0,009 | 0,16 | 0,007 | 0,19 | 0,007 |  |  |
| normalized total SV # | 25,7 | 0,9 | 18,7 | 0,7 | 19,7 | 0,6 | 18,3 | 0,6 | 7,6 | 0,7 | 5,8 | 0,4 | 5,1 | 0,5 | 5,8 | 0,4 |  |  |
| normalized docked SV # | 2,7 | 0,2 | 1,8 | 0,1 | 1,7 | 0,1 | 2 | 0,1 | 0,4 | 0,1 | 0,2 | 0,1 | 0,3 | 0,1 | 0,3 | 0,1 |  |  |
| normalized LV # | 0,5 | 0,1 | 0,8 | 0,1 | 0,5 | 0,1 | 0,9 | 0,1 | 0,6 | 0,1 | 2 | 0,2 | 1,9 | 0,2 | 0,8 | 0,1 |  |  |
|  |  |  |  |  |  |  |  |  |  |  |  |  |  |  |  |  |  |  |
|  |  |  |  |  |  |  |  |  |  |  |  |  |  |  |  |  |  |  |
| **Figure 8** | one way ANOVA | Tukey correction | |  | *, p≤0.05 | **, p≤0.01 | ***, p≤0.001 |  | all stimulated with 30s blue light | | all strains are with ChR2(H134R) | |  |  |  |  |  |  |
|  |  |  |  |  |  |  |  |  |  |  |  |  |  |  |  |  |  |  |
|  | **WT;zxIs6 -ATR** | | **WT;zxIs6 +ATR** | | **WT;zxIs6 +ATR @30°C** | | **chc-1;zxIs6 +ATR @15°C** | | **chc-1;zxIs6 -ATR @30°C** | | **chc-1;zxIs6 +ATR @30°C** | |  |  |  |  |  |  |
| N (synaptic profiles) | 89 | | 121 | | 109 | | 73 | | 88 | | 67 | |  |  |  |  |  |  |
| number of worms analyzed | 3 | | 2 | | 2 | | 2 | | 2 | | 2 | |  |  |  |  |  |  |
| number of synapses analyzed | 15 | | 19 | | 17 | | 11 | | 13 | | 12 | |  |  |  |  |  |  |
|  | **mean** | **SEM** | **mean** | **SEM** | **mean** | **SEM** | **mean** | **SEM** | **mean** | **SEM** | **mean** | **SEM** |  |  |  |  |  |  |
| area | 0,14 | 0,005 | 0,17 | 0,006 | 0,18 | 0,007 | 0,19 | 0,009 | 0,14 | 0,004 | 0,21 | 0,015 |  |  |  |  |  |  |
| normalized LV # | 0,5 | 0,1 | 0,8 | 0,1 | 0,8 | 0,1 | 0,5 | 0,1 | 1 | 0,1 | 1,2 | 0,2 |  |  |  |  |  |  |
|  |  |  |  |  |  |  |  |  |  |  |  |  |  |  |  |  |  |  |
|  |  |  |  |  |  |  |  |  |  |  |  |  |  |  |  |  |  |  |
| **Figure 10** | one way ANOVA | Tukey correction | |  | *, p≤0.05 | **, p≤0.01 | ***, p≤0.001 |  | all stimulated with 30s blue light | | all strains are with ChR2(H134R) | |  |  |  |  |  |  |
|  |  |  |  |  |  |  |  |  |  |  |  |  |  |  |  |  |  |  |
|  | **chc-1;zxIs6 +ATR @15°C** | | **chc-1;zxIs6 -ATR @30°C** | | **chc-1;zxIs6 +ATR @30°C** | | **chc-1;erp-1;zxIs6 +ATR @15°C** | | **chc-1;erp-1;zxIs6 -ATR @30°C** | | **chc-1;erp-1;zxIs6 +ATR @30°C** | | **unc-57;chc-1;zxIs6 +ATR @15°C** | | **unc-57;chc-1;zxIs6 -ATR @30°C** | | **unc-57;chc-1;zxIs6 +ATR @30°C** | |
| N (synaptic profiles) | 73 | | 88 | | 67 | | 71 | | 68 | | 61 | | 75 | | 72 | | 67 | |
| number of worms analyzed | 2 | | 2 | | 2 | | 2 | | 2 | | 2 | | 2 | | 2 | | 2 | |
| number of synapses analyzed | 11 | | 13 | | 12 | | 13 | | 12 | | 10 | | 13 | | 9 | | 10 | |
|  | **mean** | **SEM** | **mean** | **SEM** | **mean** | **SEM** | **mean** | **SEM** | **mean** | **SEM** | **mean** | **SEM** | **mean** | **SEM** | **mean** | **SEM** | **mean** | **SEM** |
| area | 0,19 | 0,009 | 0,14 | 0,004 | 0,21 | 0,015 | 0,13 | 0,007 | 0,14 | 0,006 | 0,12 | 0,006 | 0,14 | 0,005 | 0,19 | 0,009 | 0,12 | 0,004 |
| normalized LV # | 0,5 | 0,1 | 1 | 0,1 | 1,2 | 0,2 | 0,9 | 0,1 | 0,5 | 0,1 | 0,6 | 0,1 | 0,8 | 0,1 | 0,7 | 0,1 | 1,7 | 0,2 |
